# PSI Photoinhibition and Changing CO_2_ Levels Initiate Retrograde Signals to Modify Nuclear Gene Expression

**DOI:** 10.3390/antiox12111902

**Published:** 2023-10-24

**Authors:** Mehmet Kılıç, Ville Käpylä, Peter J. Gollan, Eva-Mari Aro, Eevi Rintamäki

**Affiliations:** Molecular Plant Biology, Department of Life Technologies, University of Turku, 20014 Turku, Finland; mehmet.kilic@utu.fi (M.K.); ville.kapyla@utu.fi (V.K.); peter.gollan@utu.fi (P.J.G.); evaaro@utu.fi (E.-M.A.)

**Keywords:** chloroplast retrograde signaling, FeS clusters, photoinhibition, photosystem I, redox imbalance

## Abstract

Photosystem I (PSI) is a critical component of the photosynthetic machinery in plants. Under conditions of environmental stress, PSI becomes photoinhibited, leading to a redox imbalance in the chloroplast. PSI photoinhibition is caused by an increase in electron pressure within PSI, which damages the iron–sulfur clusters. In this study, we investigated the susceptibility of PSI to photoinhibition in plants at different concentrations of CO_2_, followed by global gene expression analyses of the differentially treated plants. PSI photoinhibition was induced using a specific illumination protocol that inhibited PSI with minimal effects on PSII. Unexpectedly, the varying CO_2_ levels combined with the PSI-PI treatment neither increased nor decreased the likelihood of PSI photodamage. All PSI photoinhibition treatments, independent of CO_2_ levels, upregulated genes generally involved in plant responses to excess iron and downregulated genes involved in iron deficiency. PSI photoinhibition also induced genes encoding photosynthetic proteins that act as electron acceptors from PSI. We propose that PSI photoinhibition causes a release of iron from damaged iron–sulfur clusters, which initiates a retrograde signal from the chloroplast to the nucleus to modify gene expression. In addition, the deprivation of CO_2_ from the air initiated a signal that induced flavonoid biosynthesis genes, probably via jasmonate production.

## 1. Introduction

Photosynthesis uses solar energy to split water molecules in photosystem (PS) II on the lumenal side of the thylakoid membrane and transfers the electrons through the photosynthetic electron transport chain (PETC) to PSI, which reduces NADP^+^ in chloroplast stroma. Electron transport through the PETC simultaneously pumps protons from the stroma into the thylakoid lumen, which activates ATP synthesis by ATP synthase. ATP and NADPH energize CO_2_ fixation in the Calvin–Benson–Bassham (CBB) cycle and other metabolic pathways in the chloroplast. During photosynthetic electron transport, reactive oxygen species (ROS) are generated as by-products in PSII and PSI, especially under stress conditions. ROS, if not properly scavenged, can cause oxidative stress, but they also operate as retrograde signals that help plants to acclimate to stressful conditions [1,2]. Alternatively, electrons can also be redirected from PSI back to the PETC via cyclic electron flow (CEF), thereby strengthening the photosynthetic control mechanism and balancing the electron transport process.

Both photosystems are prone to photoinhibition, which can induce damage to the PS reaction centers, particularly in changing environmental conditions of their natural growth habitats. Under stress conditions, photoinhibition mostly impacts PSII, which in turn protects PSI from photodamage [3,4]. In chilling-sensitive plants such as cucumber, PSI photoinhibition (PSI-PI) occurs under illumination at low (4 °C) temperatures [5,6]. The degradation and replacement of the damaged PSI complex with newly synthesized PSI subunits take considerably longer than the repair of damaged PSII, which has its own dedicated repair mechanisms [3,6,7].

Upon illumination, the charge separation in PSI produces an oxidized electron donor, P700^+^, and a reduced primary electron acceptor, A_0_^−^, a chlorophyll a molecule that further transfers the electron to phylloquinone (A_1_). To compensate for the loss of an electron, the oxidized P700^+^ is reduced by plastocyanin (PC) on the lumenal side of the thylakoid membrane. A_1_ donates the electron to an iron–sulfur (FeS) cluster, FeS_X_, and then via FeS_A_ and FeS_B_ to stromal soluble ferredoxin (FD). The FeS clusters of PSI are susceptible to damage by reactive oxygen species (ROS) under stress conditions, caused by a lack of sufficient oxidized stromal electron acceptors upon excessive electron arrival from PSII [8,9]. The donation of electrons from over-reduced PSI to O_2_ leads to the production of superoxide (O_2_^•−^), which is converted into hydrogen peroxide (H_2_O_2_). H_2_O_2_ reacts with FeS clusters, leading to PSI photoinhibition and the formation of hydroxyl radicals through the Fenton reaction [10,11,12]. Hydroxyl-radical-induced damage has been shown to target the PSI core subunits PsaA and PsaB [9,13,14].

Irreversible PSI damage and a long repair process impact the redox state in chloroplasts, which may initiate a signaling cascade from the chloroplast to the nucleus to modulate gene expression for the protection and readjustment of chloroplast functions. In addition to extensively investigated chloroplast retrograde signaling during photomorphogenic development [15,16], several other chloroplast signaling pathways initiated by ROS, hormones, sugars, or redox imbalances within PETC and its electron sinks have been shown to operate in chloroplasts and relay information to adjust nuclear gene expression and initiate stress responses in plant leaves [17,18]. Besides ROS, redox imbalances in chloroplasts also lead to the generation of reactive electrophile species (RES), which can initiate signals via reactions with cellular proteins and lipids. Photosynthetic light reactions generate singlet oxygen (^1^O_2_), mainly in PSII, and PSI produces O_2_^•−^, which can be converted to H_2_O_2_ with a longer lifetime. In addition to the generation of ROS in photosynthetic light reactions, photorespiration also produces H_2_O_2_ in peroxisomes, and this reaction is enhanced by decreasing the CO_2_ concentration. The production of ROS by the PETC can trigger the synthesis of oxylipins, such as 12-oxo-phytodienoic acid (OPDA) and jasmonic acid (JA). OPDA is a type of RES and can oxidize lipids and proteins due to its reactive cyclopentane ring [19,20]. OPDA is detoxified by glutathionylation but can also be enzymatically converted to JA [21]. OPDA and JA have been shown to initiate signals that modify gene expression in the nucleus [22,23].

The likelihood of PSI photoinhibition can be mitigated by several mechanisms that reduce electron pressure within PSI, either by downregulating electron flow to the donor side of PSI or by increasing the capacity on the acceptor side. Such protective mechanisms in the former case include PSII photoinhibition, non-photochemical quenching (NPQ), and the photosynthetic control of electron transport through cytochrome b6f, while the latter includes CEF, the water–water cycle, the CBB cycle, and photorespiration [8,24], with the CBB cycle being the major sink for electrons from PSI. Therefore, we hypothesized that increasing the CO_2_ concentration upon PSI photoinhibition (PSI-PI) treatment could protect PSI against damage by providing more electron sinks, whilst decreasing the CO_2_ concentration would expose PSI to greater damage and originate a retrograde signal from chloroplasts to the nucleus to alleviate the consequences of PSI damage. To this end, we tested whether the putative production of ROS on the PSI acceptor side, oxidized lipids via the production of OPDA and JA, or damage to the FeS clusters in PSI could initiate a signaling cascade to change nuclear gene expression toward the photoprotection of PSI.

## 2. Materials and Methods

### 2.1. Growth Conditions and Light Treatments

**Figure 1:**
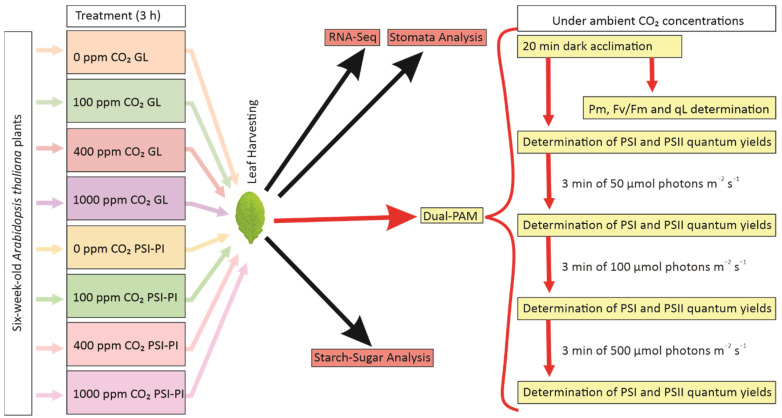
Experimental design to investigate the effects of PSI photoinhibition treatment on photosynthetic activity and gene expression at different CO_2_ concentrations. Six-week-old *Arabidopsis thaliana* plants were treated for 3 h with PSI photoinhibition (PSI-PI) treatment or with growth light (GL) as a control. The leaves of treated plants were harvested for the analyses of photosynthesis (Dual-PAM), gene expression (RNA-Seq), stomatal function, and sugar/starch content. Dual-PAM measurements were carried out at atmospheric CO_2_. After dark acclimation for 20 min, the maximum oxidation of P700 (Pm), the photochemical efficiency of PSII (Fv/Fm), and a fraction of functional PSII centers (qL) were measured in 8 leaves. For the determination of quantum yields of photosystems, the leaves were subsequently illuminated at 25, 50, 100, and 500 μmol photons m^−2^ s^−1^, each for 3 min.

Wild-type *Arabidopsis thaliana* (Columbia ecotype) was grown in a mixture of soil/vermiculite (2:1) under an 8 h photoperiod at 100 µmol photons m^−2^ s^−1^ (GL) with POWERSTAR HQI-T 400 W/D metal halide lamps (OSRAM GmbH, Munich Germany) as the light source at 23 °C and 60% relative humidity. Six-week-old plants were used for experiments. Plants were moved from growth conditions to the treatment chamber at 11 a.m. and were allowed to stabilize for 10 min in chamber conditions prior to the specific PSI-PI light treatment. A single PSI-PI treatment cycle [25] consisted of 30 s of growth light followed by three repeated cycles of 5 s of red light (660 nm, 35 µmol photons m^−^^2^ s^−1^) and 1 s of intense white light (1000 µmol photons m^−^^2^ s^−^^1^) using programmable LED lamps (Heliospectra, Göteborg, Sweden). The PSI-PI cycles were repeated for 3 h. Control plants were treated with GL intensity. Plants were subjected to PSI-PI treatment or to GL at CO_2_ concentrations of 0 ppm, 100 ppm, 400 ppm, and 1000 ppm using an air-tight chamber (Figure 1). 

### 2.2. Biophysical Measurements

The Dual-PAM-100 (Walz, Effeltrich, Germany) was used to simultaneously measure the chlorophyll a fluorescence and P700-oxidation signal in detached leaves. Before the measurements, the leaves were incubated in darkness for 20 min. The photochemical efficiency of PSII was recorded as a ratio of variable to maximum fluorescence (Fv/Fm). The maximum oxidation of P700 (Pm) was determined after far-red illumination, followed by a saturating pulse. The fraction of open PSII reaction centers in the lake model (qL) was determined following the methodology outlined by Kramer et al. [26]. The calculation involved estimating the minimum fluorescence in light (F0’) using the approach proposed by Oxborough et al. [27]. Statistical significance between different samples was tested using a one-way ANOVA with the post hoc Tukey HSD Calculator (https://astatsa.com/OneWay_Anova_with_TukeyHSD/ accessed on 1 November 2022).

For the determination of the quantum yields of photosystems, the leaves were subsequently illuminated at 25, 50, 100, and 500 μmol photons m^−2^ s^−1^ for 3 min before the measurements. The yield of functional PSI centers (Y(I)), the yield of the donor-side limitation (Y(ND)), and the yield of the acceptor-side limitation (Y(NA)) were calculated by normalizing the Pm for each leaf sample using the average Pm value of the GL samples at 400 ppm CO_2_ as a reference Pm (PmR). This normalization procedure effectively eliminates the contribution of damaged PSI reaction centers to Y(I), Y(ND), and Y(NA) [28]. The photochemical quantum yield of PSII (Y(II)), the yield of non-photochemical quenching (Y(NPQ)), and the yield of nonregulated energy dissipation (Y(NO)) were calculated following the methodology proposed by Genty et al. [29].

### 2.3. RNA Isolation, Sequencing, and Data Analyses

The seventh plant leaf was used for RNA extraction with four biological replicates. RNA was isolated using an innuPREP plant RNA isolation kit (Analytik Jena) according to the manufacturer’s instructions. The total RNA was then converted to cDNA with the Biorad iScript cDNA Synthesis Kit according to their instructions. The cDNA samples were sent to the BGI Europe Genomic Center (Copenhagen, Denmark) for sequencing. 

The raw sequence reads from each replicate were quantified with Salmon (v0.12) [30] software using *Arabidopsis thaliana* genome assembly TAIR10 cDNA sequences for the index. The transcript-level estimates from the Salmon software output were imported using ‘tximport’ R package (v3.16) and subsequently aggregated to the gene level [31]. Analyses of differential gene expression were carried out with the Bioconductor DESeq2 R package (v3.16) [32]. Genes with combined read counts lower than 10 were eliminated before differential expression analyses were performed. Genes with −1 ≥ log 2 (FC) ≥ 1 were selected for gene enrichment analyses. Gene enrichment analysis was performed with http://geneontology.org/ (accessed on 10 January 2023) software [33,34,35]. The gene lists for heatmaps were compiled manually based on GO terms or the published literature, and the heatmaps were created using the Pheatmap R package (v1.0.12). 

### 2.4. Sugars and Starch Analysis

The eighth leaves from the *Arabidopsis thaliana* plants were harvested, flash-frozen in liquid N_2_, and then ground with a bead beater for measurements of starch, sucrose, D-fructose, and D-glucose. Measurements were carried out with Megazyme total starch and Sucrose/D-Fructose/D-Glucose assay kits (K-SUFRG; Megazyme, Wicklow, Ireland) according to the manufacturer’s instructions. Statistical significance between different samples was tested using a one-way ANOVA with the post hoc Tukey HSD Calculator (https://astatsa.com/OneWay_Anova_with_TukeyHSD/ accessed on 1 May 2023).

### 2.5. Stomatal Aperture Measurement

Four leaves per PSI-PI treatment were detached from the plants and immediately imprinted on Affinis Precious Polyvinylsiloxane resin (Coltene, Cuyahoga Falls, OH, USA). The resulting negative imprints of the bottom side of each leaf were coated with quick-dry nail polish, and the thin transparent layers were observed under a microscope. From each leaf, approximately 40 stomata were imaged at 40× magnification using the EVOS M5000 Imaging System (Thermo Fischer Scientific, Waltham, MA, United States). The width and length of stomatal apertures were measured using ImageJ software (Fiji, v2.9.0), and the stomatal aperture index (SAI) was calculated as width per length. 

## 3. Results

### 3.1. Susceptibility of PSI to Specific Photoinhibition Treatment Is Almost Independent of CO_2_ Concentration

**Figure 2:**
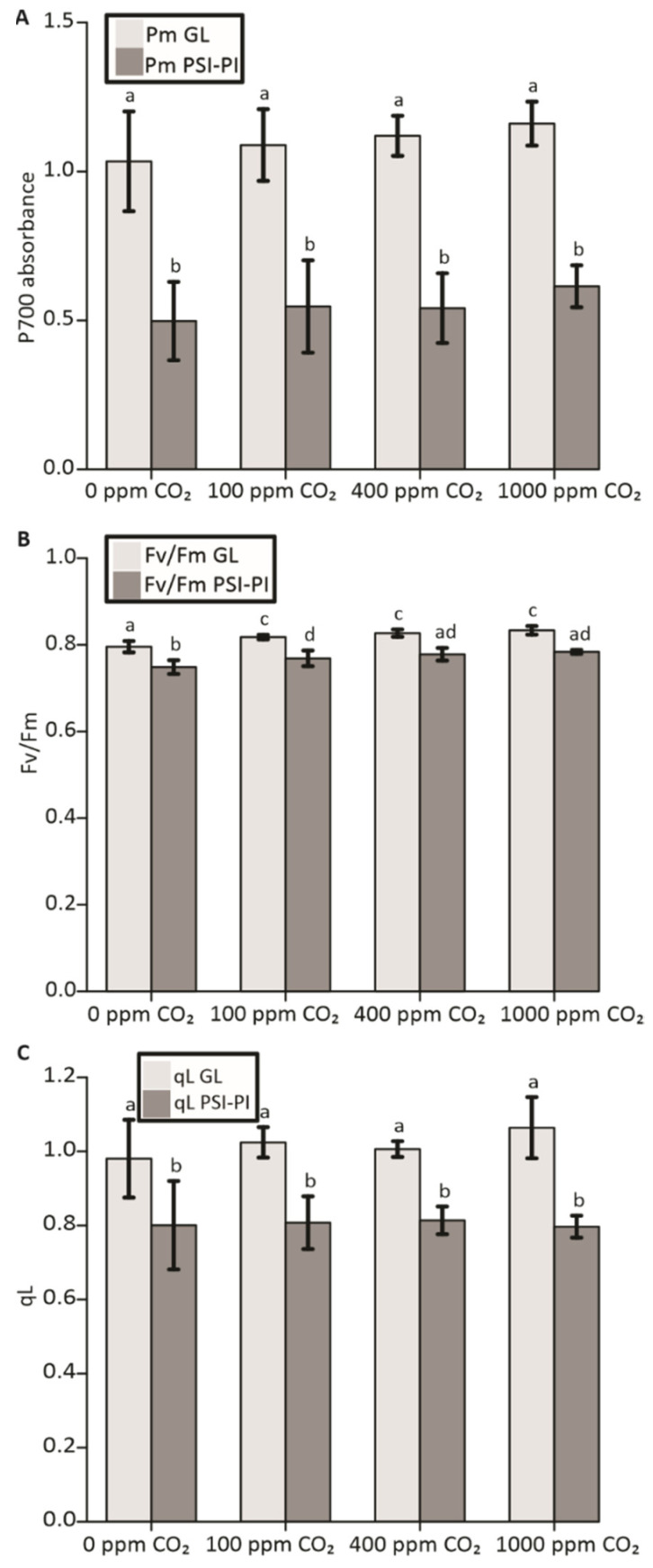
Photosynthetic parameters in *Arabidopsis* plants treated with growth light (GL; light gray) or with PSI photoinhibition (PSI-PI; dark gray) treatment at various CO_2_ concentrations. Light treatments were carried out for three hours at CO_2_ concentrations of 0 ppm, 100 ppm, 400 ppm, and 1000 ppm. Principal photosynthetic parameters were measured with the Dual-PAM 100 (WALZ) on 8 leaves after dark adaption for 20 min. (**A**) Maximum oxidation of P700 (Pm). (**B**) Maximum quantum efficiency of PSII photochemistry (Fv/Fm). (**C**) Fraction of functional PSII centers in dark-acclimated samples (qL). Error bars represent standard deviations, and the letters indicate significant differences between the treatments (ANOVA, Tukey HSD, *p* < 0.05).

As summarized in Figure 1, plants were exposed to either the PSI-PI treatment, which induces PSI photoinhibition, or to GL for three hours at a CO_2_ concentration of 0 ppm, 100 ppm (low), 400 ppm (atmospheric), or 1000 ppm (high). After the treatments, leaves were harvested for the analyses of gene expression (RNA-Seq), sugar quantity, and stomatal aperture, as well as for the determination of photosynthetic parameters after 20 min of dark incubation. Measurements of PSII fluorescence parameters and the maximum oxidation of PSI were recorded at atmospheric CO_2_. The PSI-PI treatment reduced the PSI oxidation capacity by about 50% at all CO_2_ concentrations tested (Figure 2). Interestingly, the absence of CO_2_ did not exacerbate PSI photoinhibition, and 1000 ppm CO_2_ concentration did not protect PSI from photoinhibition (Figure 2A). The PSI-PI treatment caused only a slight decrease in the Fv/Fm of PSII (Figure 2B) and an approximately 20% decrease in qL (Figure 2C) at all CO_2_ conditions.

**Figure 3:**
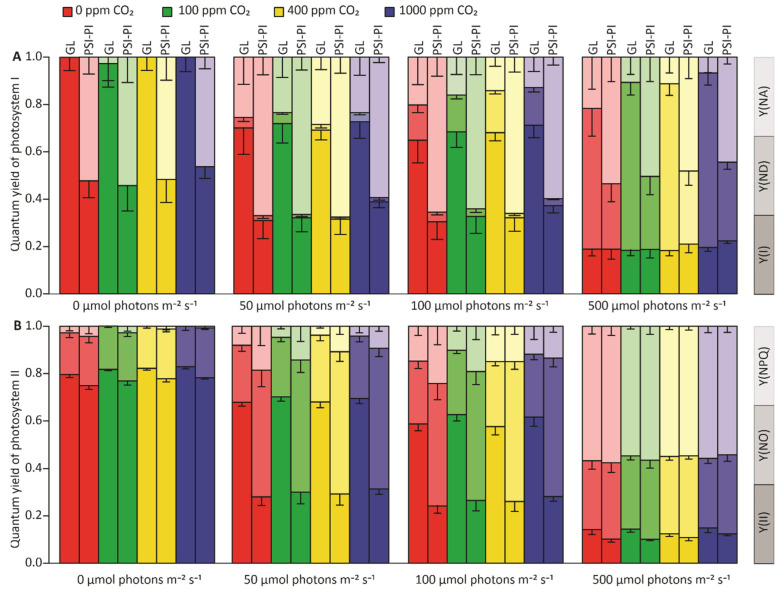
PSI and PSII quantum yields in *Arabidopsis* leaves illuminated under growth light (GL) or treated with PSI photoinhibition (PSI-PI) treatment at various CO_2_ concentrations. GL plants were treated with growth light (100 μmol photons m^−2^ s^−1^), and PSI-PI plants were treated with PSI photoinhibition light for three hours at CO_2_ concentrations of 0 ppm, 100 ppm, 400 ppm, and 1000 ppm. After illumination, plants were dark-acclimated for 20 min and subsequently illuminated at 0, 25, 50, 100, and 500 μmol photons m^−2^ s^−1^ for 3 min with Dual-PAM 100 for measurements of the quantum yield of functional photosystems. (**A**) PSI quantum yields: PSI photochemistry (Y(I)), PSI-donor-side limitation (Y(ND)), and PSI-acceptor-side limitation (Y(NA)). (**B**) PSII quantum yields: PSII photochemistry (Y(II)), nonregulated energy dissipation in PSII (Y(NO)), and regulated non-photochemical energy dissipation in PSII (Y(NPQ)). Error bars represent standard deviations (*p* < 0.05, n = 8).

Plants exposed to either the PSI-PI or GL treatment at different CO_2_ concentrations were subjected to measurements of the quantum yields of PSI and PSII at different light intensities (Figure 3). The quantum yields of PSI, indicative of PSI photochemistry (Y(I)), were calculated for the remaining functional PSI centers after the PSI-PI treatment (see Materials and Methods). The quantum yields of functional PSI centers were approximately 50% lower in PSI-PI-treated plants measured at light intensities of 0, 50, and 100 µmol photons m^−^*^2^* s^−1^ in comparison to control plants without PSI-PI treatment (Figure 3A). Under high light, Y(I) of PSI-PI-exposed plants did not differ from that of control plants (Figure 3A). As expected, the acceptor-side limitation (Y(NA)) of the functional PSI centers in PSI-PI plants was substantially higher in all conditions compared to control plants (Figure 3A). PSI-PI treatment induced an approximately 50% decrease in PSII photochemistry (Y(II)) and corresponding increases in nonregulated energy dissipation (Y(NO)) and regulated energy dissipation (Y(NPQ)) in plants illuminated at light intensities of 50 and 100 µmol photons m^−^*^2^* s^−1^, whereas only minor differences in Y(II), Y(NO) and Y(NPQ) were detected between PSI-PI-treated and control leaves at 0 and 500 µmol photons m^−^*^2^* s^−1^ (Figure 3B). These results indicated that the PSI-PI treatment impaired the light energy utilization of the PETC under light intensities, limiting photosynthesis. Importantly, however, changes in CO_2_ concentration had very little effect on the quantum yields of PSI and PSII in both GL- and PSI-PI-treated plants.

### 3.2. Effects of Combined PSI-PI and Different CO_2_ Treatments on Carbohydrate Accumulation and Stomatal Opening

To analyze the effect of PSI photoinhibition on the accumulation of photosynthetic carbon metabolites in leaves, we measured the starch, fructose, glucose, and sucrose contents, as well as the stomatal aperture, in GL- and PSI-PI-treated leaves. 

**Figure 4:**
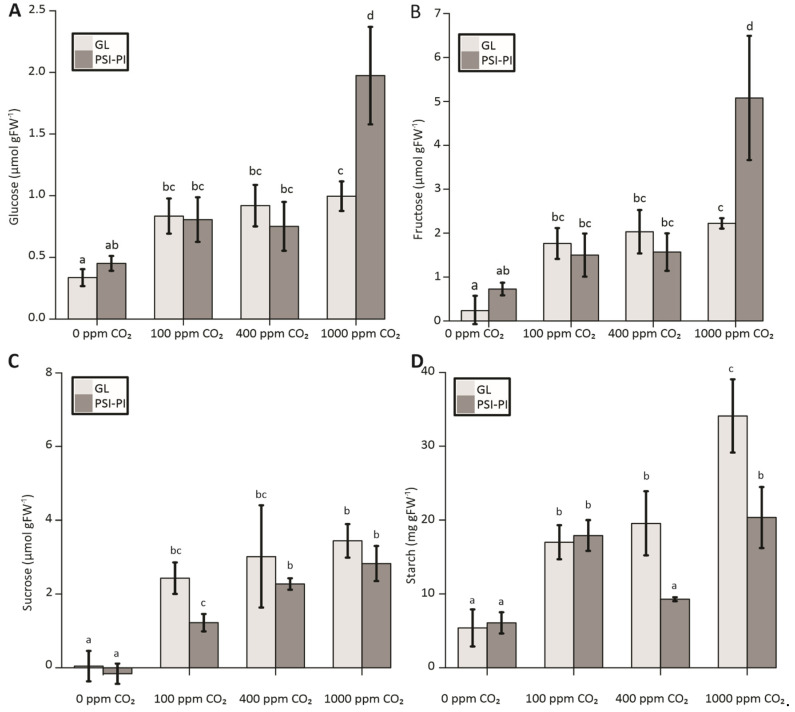
Starch and sugar contents in *Arabidopsis* leaves treated with either growth light of 100 μmol photons m^−2^ s^−1^ (GL; light gray) or with PSI photoinhibition (PSI-PI; dark gray) treatment for three hours at CO_2_ concentrations of 0 ppm, 100 ppm, 400 ppm, and 1000 ppm. The quantification of starch and sugars was carried out with Megazyme assay kits and expressed as µmol per g fresh weight (FW) for glucose (**A**), fructose (**B**), and sucrose (**C**) and as mg per g FW for starch (**D**). SE, error bars (n = 5). Bars with different letters indicate significant differences with a *p*-value < 0.05 (ANOVA, Tukey HSD).

Compared to GL plants, PSI-PI treatment did not significantly reduce the accumulation of glucose or fructose in leaves exposed to atmospheric or lower CO_2_ concentrations, whereas PSI-PI-treated leaves had significantly higher amounts of these sugars at a high CO_2_ of 1000 ppm (Figure 4A,B). The amount of sucrose was slightly, but not significantly, lower in PSI-PI-treated leaves compared to GL plants (Figure 4C). PSI-PI treatment had no effect on starch accumulation in leaves exposed to 0 or 100 ppm CO_2_, whereas about 40 to 50% reduction in starch content was observed in PSI-PI-treated plants at 400 and 1000 ppm CO_2_ (Figure 4D).

As it has been reported that leaf stomatal closure responds to CO_2_ concentrations [36], we made leaf imprints and calculated the stomatal aperture index (width/length), which is indicative of stomatal conductance, after GL or PSI-PI exposure to various CO_2_ concentrations (Figure S1). The stomatal aperture index was slightly but significantly higher in PSI-PI leaves in the absence of CO_2_. At 1000 ppm CO_2_, the stomatal aperture was significantly lower in both the control and PSI-PI-treated leaves compared to all other conditions.

### 3.3. Differential Gene Expression Induced by Exposure of Plants to PSI-PI Treatment and Different CO_2_ Concentrations

#### 3.3.1. Changes in Gene Expression Induced by Varying CO_2_ Concentrations

**Figure 5:**
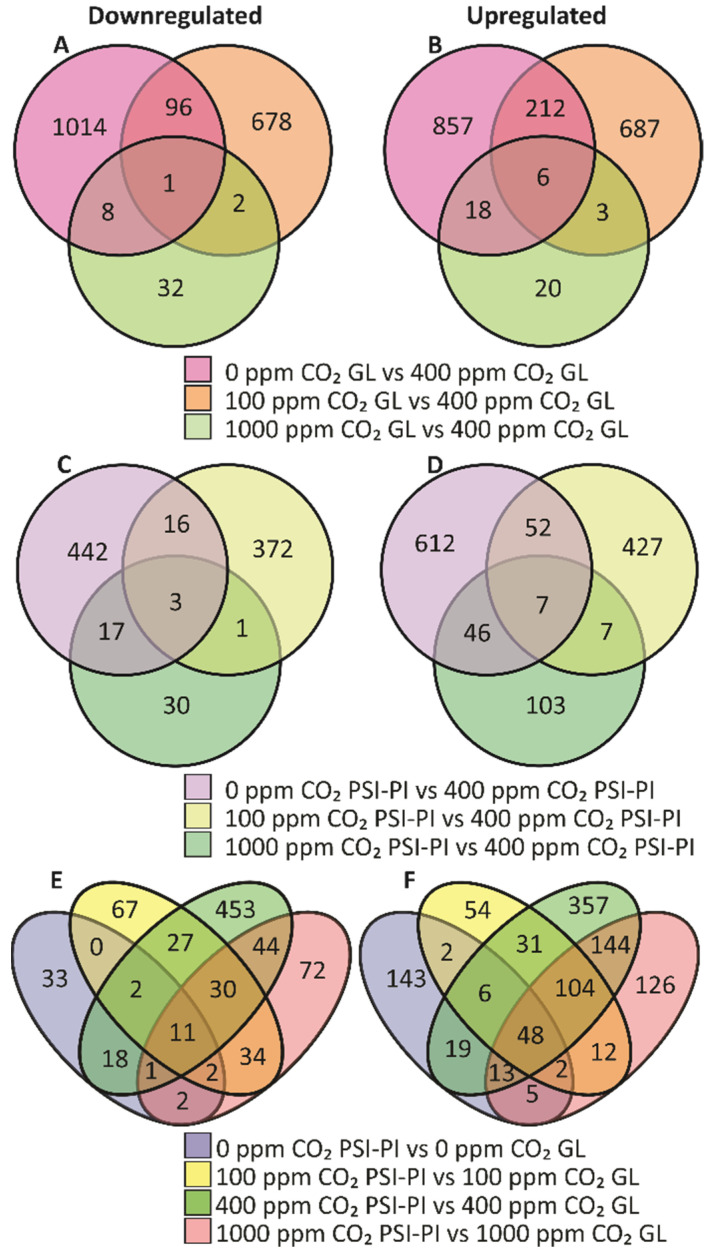
Venn diagrams of differentially expressed genes in plants treated either with growth light (GL) or with PSI photoinhibition (PSI-PI) at CO_2_ concentrations of 0 ppm, 100 ppm, 400 ppm, and 1000 ppm. Downregulation (**A**) and upregulation (**B**) of genes in leaves exposed to different CO_2_ concentrations at GL in comparison to plants exposed to 400 ppm (control) CO_2_. Downregulation (**C**) and upregulation (**D**) of genes in leaves exposed to PSI-PI treatment at different CO_2_ concentrations in comparison to plants exposed to PSI-PI treatment at 400 ppm CO_2_. Downregulation (**E**) and upregulation (**F**) of genes in leaves exposed to PSI-PI treatment at different CO_2_ concentrations in comparison to plants without PSI-PI treatment but at the same CO_2_ concentration. The genes with −1 ≥ log2 ≥ 1 expression fold change with respect to relative controls and with a *p*-value < 0.05 are included in the figure. The list of the genes is presented in the Table S1.

First, we focused on differentially expressed genes (DEGs) in leaves exposed to CO_2_ reduction and elevation relative to atmospheric CO_2_ (Figure 5A–D). Abnormal CO_2_ concentrations caused major changes in gene expression relative to 400 ppm CO_2_, with only 10% overlap between the CO_2_ treatments in GL-treated plants (Figure 5A,B) and 7% overlapping genes between the CO_2_ treatments in PSI-PI-treated plants (Figure 5C,D). A minority (21% at 0 and 100 ppm; 7% at 1000 ppm) of the genes were differentially expressed in both the GL- and PSI-PI-treated plants (Figure S2).

The deprivation of CO_2_ from the air induced a large number of DEGs with high fold-changes (log2 > 4) (Table S1). Interestingly, many of these genes were upregulated in both GL- and PSI-PI-treated leaves (Table S1), indicating that the response was specifically related to CO_2_ removal. Furthermore, the most highly induced genes included many involved in the biosynthesis of secondary metabolites (flavonoids, anthocyanins) derived from phenylalanine (Table 1). In total, we found 20 upregulated genes involved in flavonoid metabolism in GL- and PSI-PI-treated leaves exposed to 0 ppm CO_2_. The genes encoding CHALCONE SYNTHASE (CHS) and DIHYDROFLAVONOL 4-REDUCTASE (DFR), key enzymes in flavonoid synthesis [37,38,39], were strongly induced at 0 ppm CO_2_. CHS catalyzes the synthesis of naringenin chalcone, which is converted to narigenin by CHALCONE ISOMERASE (CHI). Naringenin is a precursor to several classes of flavonoids. DFR converts dihydroflavonols to flavan-3-ols, which serve as precursors for the synthesis of anthocyanins. The expression of the other genes encoding enzymes involved in flavonoid biosynthesis (UF3GT, AT5MAT, LDOX) or metabolism (GSTF12) was also upregulated at 0 ppm CO_2_. Accordingly, CO_2_ deprivation induced eight genes encoding MYB transcription factors (Table 1), which have previously been shown to regulate the biosynthesis of flavonoids [37,39,40]. Moreover, the bHLH, WRKY, and NAC transcription factors (TT8, GL3, TTG2, NAM, NAC032) (Table 1) are also involved in the regulation of the expression of flavonoid biosynthesis genes [37,39].

Low CO_2_ (100 ppm) also significantly altered the gene expression, with 1685 DEGs in GL-treated plants and 885 DEGs in PSI-PI-treated plants compared to atmospheric CO_2_ (Figure 5A–D). In contrast to the response to 0 ppm CO_2_, the number of overlapping DEGs between GL and PSI-PE treatments was minimal (Table S1). Elevated (1000 ppm) CO_2_ only slightly altered the gene expression in GL plants compared to atmospheric CO_2_. The fold change in DEGs was also lower than in leaves exposed to 0 and 100 ppm CO_2_ (Table S1).

All combinations of CO_2_ and PSI-PI treatments resulted in a very low number of overlapping DEGs. Interestingly, however, the small number of DEGs upregulated in both GL- and PSI-PI-treated leaves at all divergent CO_2_ concentrations included three genes encoding cytosolic and chloroplast COPPER/ZINC SUPEROXIDE DISMUTASE and a COPPER CHAPERONE for copper–zinc superoxide dismutase (Table S1). This suggested that superoxide scavenging may be an important function under the stress induced by changes in CO_2_ concentration.

#### 3.3.2. PSI-PI Treatment Induced Differential Expression of Unique Genes Involved in Iron Homeostasis and Light Receptor Signaling

Next, we analyzed changes in gene expression induced by PSI photoinhibition by identifying the DEGs in PSI-PI-treated leaves compared to GL-treated leaves at identical CO_2_ concentrations (Figure 5E,F). As with the variation in the CO_2_ concentration, the change in gene expression was mainly unique for each CO_2_ level, with only 30% of the total DEGs overlapping. Interestingly, the highest number of PSI-PI-induced DEGs was observed in leaves treated with 400 ppm CO_2_. Furthermore, the highest number of overlapping DEGs (10%) was found in leaves treated with 400 ppm and 1000 ppm CO_2_, indicating that elevated CO_2_ less drastically modified leaf metabolism than CO_2_ deprivation, as is also evident in Figure 5A,B.

Fifty-nine genes differentially expressed in PSI-PI-treated plants at all CO_2_ concentrations tested contained a high proportion of genes involved in iron (Fe) homeostasis (Table 2). Taking all CO_2_ treatments together, twenty-two genes encoding proteins involved in Fe metabolism were differentially expressed in PSI-PI-treated leaves (Table 2). Genes encoding leaf-type and chloroplast-localized FERRITIN proteins (FER1, 3, and 4) were significantly upregulated in PSI-PI-treated leaves (Table 2). FERs are iron storage proteins that are induced by a local or temporal excess of Fe content in plants [41,42]. In addition to the *FER*s, eleven other Fe metabolism genes were upregulated by PSI-PI treatment (Table 2), including *NEET*, *FRO1*, *6*, and *7*, and *VTL1* and *2* genes, encoding membrane proteins that are involved in the transportation of Fe or FeS clusters between the cytosol and organelles [43,44]. The upregulated genes *CYTOSOLIC HEME BINDING PROTEIN1*, *2*, and *3* (*cHBP*) encode tetrapyrrole-binding proteins [45], and the expression of *ENH1* and *AT3G49160* genes has been shown to be downregulated in response to iron deficiency [46,47]. On the other hand, the PSI-PI treatment repressed eight genes previously shown to be induced by Fe deficiency in plants (Table 2). The prominent upregulation of *FER* and Fe transporter genes and the concomitant repression of Fe-deficiency-induced genes in PSI-PI-treated leaves suggest that PSI photoinhibition causes the release of iron from PSI.

The DEGs regulated by PSI-PI treatment also included genes encoding light-signaling components with upregulated (*HY5*, *HYH*, *COL2*, *ARF*, *AT5G18404*, *SIG5*) or repressed (*RPGE1*, *3*, and *4*; *PIL1* and *2*; *SIB1*) expression (Table 3). HY5 protein acts downstream from light receptors during photomorphogenesis of seedlings (for reviews, see, e.g., [48,49]). HYH is homologous to HY5 [50]. *CONSTANS-LIKE 2* (*CON2*), *ATTENUATED FAR-RED RESPONSE* (*ARF*), and *AT5G18404* belong to the phytochrome (Phy) signaling network [51,52], and SIGMA FACTOR 5 (SIG5) controls chloroplast gene expression [53]. Besides the upregulation of light-signaling components, PSI-PI treatment significantly reduced the expression of genes encoding suppressors of light signaling (Table 3).

#### 3.3.3. PSI-PI Treatment Upregulates Genes Encoding the Components of Cyclic Electron Flow, CBB Cycle, and Photorespiration

**Figure 6:**
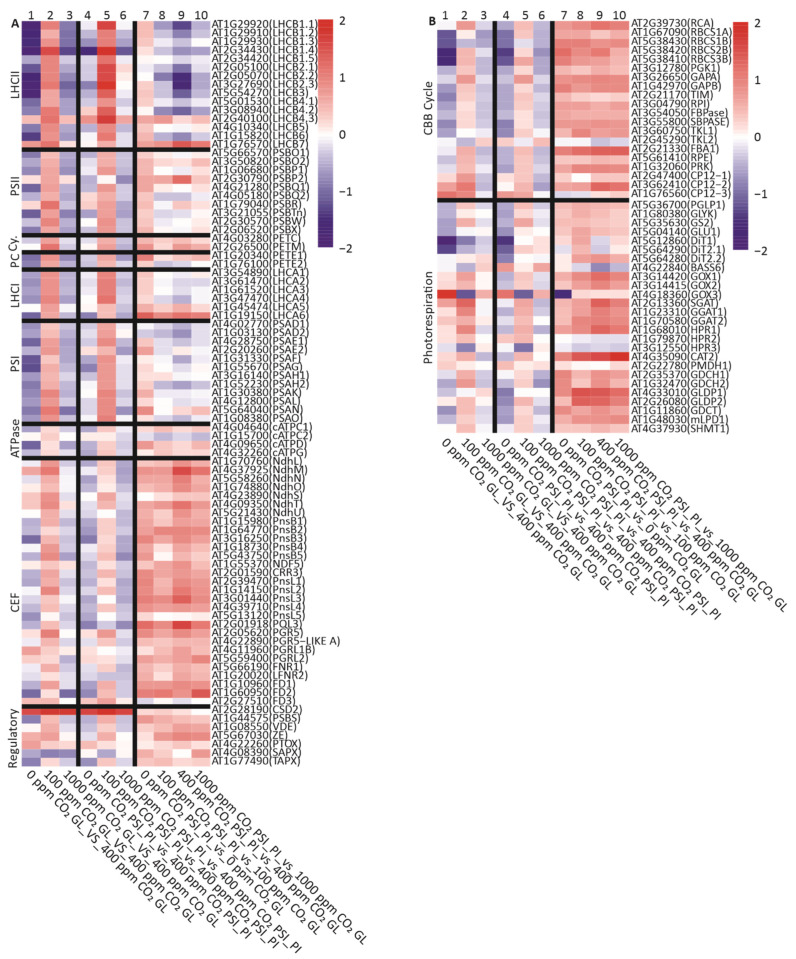
Differential expression of nuclear genes encoding proteins involved in (**A**) photosynthetic electron transport chain (PETC) and (**B**) Calvin–Benson–Basshan (CBB) cycle and photorespiration pathways. The heatmap shows the log2 values of differentially expressed genes in plants exposed to growth light (GL) and specific PSI photoinhibition (PSI-PI) treatment for three hours at various CO_2_ concentrations. The treatments compared in the columns are indicated under the figure. Columns: 1–3, changes induced by abnormal CO_2_ concentrations under constant growth light; 4–6, changes induced by abnormal CO_2_ concentrations in PSI-PI- treated plants; 7–10, changes induced by PSI-PI treatment at various CO_2_ concentrations indicated in the figure. The gene lists were compiled manually to include all nuclear genes encoding PETC subunits as well as CBB cycle and photorespiratory enzymes (Table S3). Cy.: Cyt*b*_6_*f*; PC: plastocyanin.

The different leaf treatments presented here induced a high number of moderate DEGs (fold change −2 < log2 < 2) (Table S1). To obtain an overall view of changes in nuclear gene expression, we conducted an enrichment analysis of DEGs to reveal the cellular functions targeted by changes in the CO_2_ concentration or by the PSI-PI treatment. Several pathways related to chloroplast metabolism emerged from the analysis: PETC, sugar metabolism, and ROS and JA signaling (Table S2). To investigate the specific effect of PSI-PI treatment on the expression of photosynthetic genes, we compared the gene expression in PSI-PI-treated leaves with the corresponding result in GL leaves at each CO_2_ concentration (columns 7 to 10 in Figure 6A). PSI-PI treatment, independently of the CO_2_ concentration, moderately upregulated the genes encoding the subunits of chloroplast NAD(P)H DEHYRDOGENASE-LIKE (NDH) complex and PROTON GRADIENT REGULATION (PGR) proteins (Figure 6A). These two complexes mediate electron flow from FD to thylakoid plastoquinone (PQ) pool via two different CEF pathways [54,55,56,57]. Accordingly, the genes encoding the leaf-type FDs (FD1 and FD2), the major photosynthetic isoforms participating in thylakoid electron transfer reactions, were upregulated by PSI-PI treatment (Figure 6A). At the same time, the root-type FD3 did not significantly respond to either PSI-PI treatment or changes in the CO_2_ concentration. The expression of the genes encoding subunits of other thylakoid photosynthetic complexes was either not significantly changed (PSII, Cytb_6_f, and ATPase) or slightly (PSI, LHCI) or moderately (LHCII) downregulated in PSI-PI-treated leaves at 100, 400, and 1000 ppm CO_2_, with the decrease being the most pronounced in PSI-PI-treated leaves at ambient CO_2_ concentration. The results in Figure 6A suggest that the PSI-PI treatment generally activates a signaling cascade that induces the expression of *CEF* genes and downregulates the expression of *LHCB* genes while having minimal effects on the expression of other PETC genes.

Next, we investigated how the expression of nuclear genes involved in the CBB cycle, photorespiration, and sugar metabolism directly derived from the CBB cycle responded to the PSI-PI treatment at different CO_2_ concentrations. PSI-PI treatment upregulated many key enzymes in the CBB cycle and photorespiration (columns 7 to 10 in Figure 6B and Figure S3), independently of the CO_2_ concentration. The CBB genes involved in the carboxylation/oxygenation of RuBP (Rubisco and Rubisco activase), in the production of triose phosphates (GAP), and in the regeneration reactions of RuBP (RPI, TKL1, and PRK) were upregulated by PSI-PI treatment. The gene encoding the second isoform of TRANSKETOLASE, TKL2, did not respond to PSI-PI treatment (Figure 6B). TKL2 has been reported to be mainly expressed in seeds and senescing leaves [58], suggesting that it is not an important enzyme for photosynthetic carbon fixation. 

In the photorespiration pathway, several genes encoding peroxisomal enzymes showed higher expression in PSI-PI-treated leaves compared to GL leaves (Figure 6B and Figure S3) [59]. The expression of the peroxisomal *CATALASE2* (*CAT2*) gene was significantly induced by PSI-PI treatment (Figure 6B). Furthermore, the expression of genes encoding enzymes that process the photorespiratory amino acid intermediates (*GGAT1*, *GGAT2*, and *SGAT*) was also upregulated in response to the PSI-PI treatment, as well as the *HPR1* gene encoding the enzyme producing glycerate in the final phase of photorespiration (Figure 6B and Figure S3). In addition to peroxisomal enzymes, the expression of genes encoding mitochondrial enzymes involved in photorespiration was also upregulated by the PSI-PI treatment, especially genes encoding enzymes that catalyze the production of serine from glycine (GLDP1, GLDP2) (Figure 6B and Figure S3).

The genes involved in the synthesis and degradation of starch and sucrose did not show any clear differential expression patterns induced by either the PSI-PI treatment or changes in the CO_2_ concentration (Figure S4). Although PSI-PI treatment reduced the accumulation of starch at atmospheric and elevated CO_2_ concentrations (Figure 3), the expression of genes encoding chloroplast starch synthesis were not significantly differentially expressed in these leaves (Figure S4).

#### 3.3.4. Search for the Origin of Regulatory Signals Generated by PSI-PI Treatment at Different CO_2_ Concentrations

**Figure 7:**
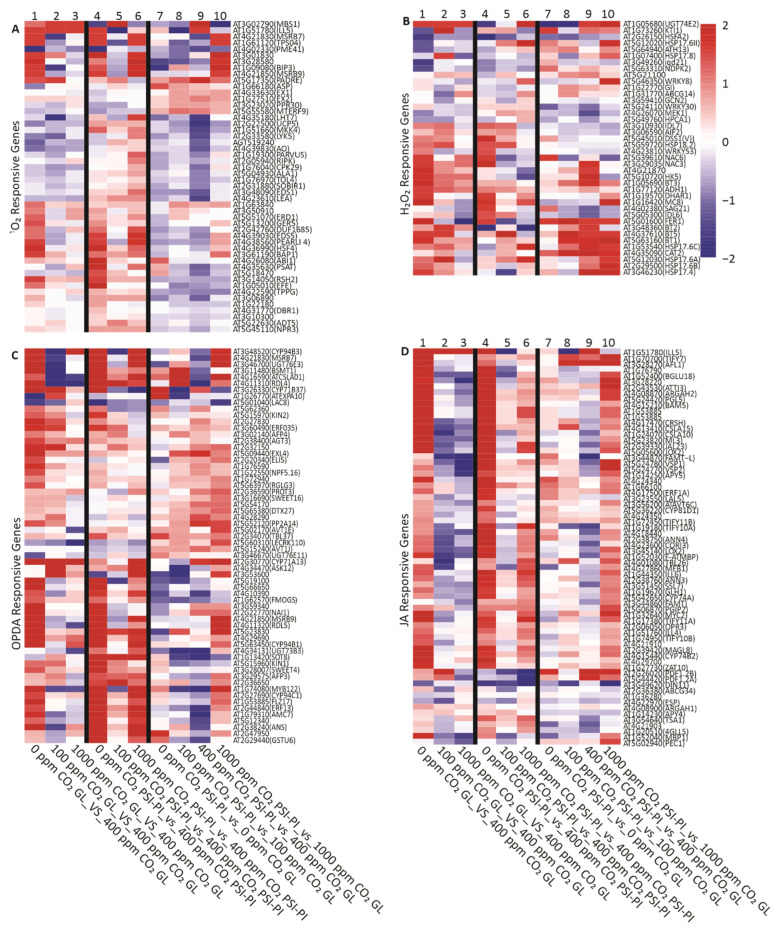
Differential expression of genes induced by various signaling molecules in *Arabidopsis thaliana* leaves exposed to GL and PSI photoinhibition (PSI-PI) treatment at CO_2_ concentrations of 0, 100, 400, and 1000 ppm. The heatmap shows the log2 values of the genes upregulated by (**A**) singlet oxygen (^1^O_2_), (**B**) hydrogen peroxide (H_2_O_2_), (**C**) 12-oxo-phytodienoic acid (OPDA), and (**D**) jasmonic acid (JA). The genes used in the analyses are listed in Table S3. The plant treatments are indicated below each column. The ^1^O_2_ gene list consists of genes reported to be ^1^O_2_-responsive in Op Den Camp et al. [64], with the addition of genes included in the following GO terms: singlet-oxygen-mediated programmed cell death (GO:0010343), response to singlet oxygen (GO:0000304), cellular response to singlet oxygen (GO:0071452). The H_2_O_2_ gene list consists of genes included in the following GO terms: response to oxidative stress (GO:0006979), hydrogen peroxide catabolic process (GO:0042744), cellular response to hydrogen peroxide (GO:0070301). The OPDA and JA gene list consists of genes reported to be OPDA- or JA-responsive in Gollan and Aro [22]. The columns are described in Figure 6.

To identify the origin of the chloroplast-generated retrograde signals produced by the PSI-PI treatment and changes in the CO_2_ concentration, we next collected the genes known from the literature to be induced by potential initiators of chloroplast signaling (^1^O_2_, H_2_O_2_, oxylipins, JA, HL, and nitric oxide (NO)) and analyzed their expression with our experimental setup (Figure 7 and Figures S5 and S6). The majority of genes known to respond to OPDA and JA were upregulated in GL- and PSI-PI-treated leaves exposed to 0 ppm CO_2_ (columns 1 and 4 in Figure 7C,D), indicating that the major signals regulating the nuclear gene expression at 0 ppm are likely to be mediated by OPDA and/or JA. Only about 50% of the genes responding to ^1^O_2_ or H_2_O_2_ were induced in GL- and PSI-PI-treated leaves exposed to 0 ppm CO_2_ (columns 1 and 4 in Figure 7A,B), suggesting that these are less likely to be the signaling molecules. The DEG profiles at 100 ppm CO_2_, with respect to 400 ppm, suggested that neither ^1^O_2_ nor JA signaling (columns 2 and 5 in Figure 7A,D) explain the change in gene expression in leaves exposed to 100 ppm CO_2_. The exposure of leaves to 1000 ppm CO_2_ did not activate any of the systematic signals analyzed in Figure 7. 

The PSI-PI treatment at 400 ppm CO_2_ did not activate either JA- or ^1^O_2_-responsive genes (column 9 in Figure 7C,D), indicating that the origin of DEGs cannot be traced to these compounds. On the other hand, only 40% of OPDA-responsive genes were slightly activated by the treatment, and only 30% of H_2_O_2_-responsive genes were moderately upregulated (column 9 in Figure 7B,C), suggesting that the signal(s) induced by the PSI-PI treatment at atmospheric CO_2_ may be only partially related to the signaling cascades induced by these compounds. 

In addition to ROS, NO has also been reported to induce *FER* gene expression, followed by Fe accumulation in plant tissues [41]. Therefore, we tested whether the NO-responsive genes [60,61] would also respond to the PSI-PI treatment (Figure S5). In general, no similar gene expression profiles typical of NO treatment were observed in PSI-PI-treated leaves (columns 7 to 10 in Figure S5). Only *FER1* was significantly induced, suggesting that the changes in nuclear gene expression in PSI-PI-treated leaves were not caused by NO signaling.

The fluctuating-light regime included in our PSI-PI treatment comprised short pulses of high-intensity light (see Materials and Methods), which may trigger the signaling cascades observed in HL-exposed leaves. We therefore compared our transcriptomic data with those published by Alvarez-Fernandez [62], where *Arabidopsis* plants were transferred from 150 to 1100 µmol photons m^−2^ s^−1^ for 3.5 h. We took the genes that were most up- and downregulated by the PSI-PI treatment (Table S1) and analyzed how the expression of these genes had changed after the 3.5 h HL treatment reported by Alvarez-Fernandez [63]. Approximately 70% of the genes most upregulated by PSI-PI treatment were also induced by HL, whereas most of the genes downregulated by PSI-PI did not respond to the HL treatment (Figure S6). The comparison of Fe homeostasis DEGs after the PSI-PI treatment with the HL treatment indicated that *FER*s, *cHBP1*, and *ENH1* were induced by HL, while most of the Fe-deficiency genes suppressed by PSI photoinhibition (Table 2) did not respond to the HL treatment (Figure S6). This indicated that although the transcript profiles after PSI-PI treatment and HL had some similarities, 50% inhibition of PSI also had a specific and partially stronger effect on nuclear gene expression than HL alone.

To gain further insight into the signaling cascade induced by the PSI-PI treatment at different CO_2_ concentrations, we used the Genevestigator database [63] and searched for experiments where light conditions were modified or the plants were treated with ROS, OPDA, JA, or elevated CO_2_, and then we analyzed the expression of PETC, CBB, and photorespiratory genes in these accessions (Figure S7). Only the extreme light change experiment, in which mature plants grown at very low light intensity were transferred to 100-times higher light intensity for 6 h, gave a similar pattern of photosynthetic gene expression to that observed in plants after the PSI-PI treatment (Figure 6): the upregulation of CEF, CBB, and photorespiratory genes and the downregulation of LHCB genes. Thus, the redox imbalance induced by PSI-PI treatment in the chloroplast resembles the conditions created by a strong increase in light intensity in the plant environment.

## 4. Discussion

### 4.1. PSI-PI Treatment of Leaves Induces Similar PSI Photoinhibition Independent of CO_2_ Concentration

In this study, we focused on the influence of PSI-acceptor-side components on the susceptibility of PSI to light damage and on the global transcriptomic changes induced by the imbalanced function of the PETC. To this end, *Arabidopsis* plants were exposed to various CO_2_ concentrations simultaneously with an artificial fluctuating-light (PSI-PI) treatment that specifically induces PSI damage with only minor effects on PSII function (Figure 2) [25]. It has been shown that PSI photoinhibition in *Arabidopsis* is caused by damage to FeS clusters in PSI [12]. Previously, we reported that PSI-PI treatment significantly decreases the CO_2_ assimilation rate [28,65]. However, as shown in Figure 2C, a similar decrease in PSI occurred independently of the CO_2_ concentration, indicating that neither the reduction nor the elevation in the CO_2_ level significantly affected the susceptibility of PSI to photoinhibition. This finding may indicate that there is no substantial influence of CO_2_ on the oxidation state of PSI in our experimental setup. It is therefore conceivable that over-reduction by PSI during the PSI-PI treatment destroys FeS clusters more rapidly than oxidized FD can accept electrons from PSI. It is also likely that the artificial PSI-PI treatment does not directly mimic any natural stress conditions inducing PSI photoinhibition, although the damage to PSI FeS clusters seems to also occur similarly during cold-induced PSI photoinhibition [66]. Nevertheless, the PSI-PI treatment is a useful tool to investigate the stress symptoms initiated by changes in the internal CO_2_ concentration of leaves and occurring concomitantly with damage to PSI. For example, a fluctuating natural light environment combined with drought may damage the function of PSI but simultaneously induce stomatal closure, thereby decreasing the internal CO_2_ concentration in leaves. Likewise, sudden cold weather during the springtime causes a decline in stromal enzyme activities but is often combined with bright daylight, causing over-reduction by the PETC and damage to PSI.

PSI-PI treatment at 400 and 1000 ppm CO_2_ had the strongest effect on leaf carbon metabolism (Figure 4). The accumulation of starch was reduced by half, whereas the content of monosaccharides (glucose and fructose) was nearly doubled at high CO_2_ (Figure 4). Yet, no differences in stomatal aperture were detected between GL- and PSI-PI-treated leaves at 400 or 1000 ppm CO_2_ (Figure S1), indicating that the lower starch content of PSI-PI-treated leaves is not due to stomatal function. Chloroplast starch synthesis is regulated by ADP-glucose pyrophosphorylase (AGPase), a redox-regulated enzyme activated under light by thioredoxins [67,68]. PSI-PI treatment, however, causes the oxidation of stromal enzymes [28], suggesting that the reduced starch synthesis in PSI-PI-treated leaves results from AGPase inactivation, with a consequent imbalance in leaf carbon metabolism. This conclusion is supported by the observation that impaired starch synthesis in chloroplasts increases the accumulation of glucose and fructose in leaves [69]. Imbalanced starch metabolism may impair plant growth for days after the termination of PSI-PI stress because, firstly, the repair of photodamaged PSI centers is a very slow process [6,7,65], and secondly, the chloroplast starch reservoirs are an important energy source for diel plant growth [70,71,72].

### 4.2. CO_2_-Specific Changes in Gene Expression: Removal of CO_2_ Activates Flavonoid Metabolism, Likely via JA/OPDA Signaling in Leaves

Genes that strongly responded to 0 ppm CO_2_ are shown to encode enzymes and transcription factors involved in metabolic pathways for the production of flavonoids (Table 1). Flavonoids are antioxidants that play a critical role in plant interactions with their environments [73], and recently, Banerjee et al. [74] demonstrated that the levels of flavonoids increase in the absence of CO_2_. Here, we found that 20 genes involved in flavonoid biosynthesis were upregulated in response to CO_2_ deprivation, including 7 biosynthetic enzymes and 13 transcription factors (Table 1). The upregulation of these *MYB* genes is likely to lead to the activation of flavonoid biosynthesis genes [37,39,75,76]. R2R3-MYB transcription factors (MYB75, MYB90, MYB113, and MYB114) form an R2R3-MYB/bHLH/WD40 complex with GL3/EGL3/TT8 and TTG1 transcription factors [77,78]. This complex specifically activates the expression of enzymes that catalyze flavonoid synthesis and anthocyanin metabolism [37,78]. 

JA mediates the accumulation of flavonoids by inducing the expression of R2R3-MYB genes and flavonoid synthesis genes [16,23,79,80]. It has been shown that JA receptor mutants exhibit a deficiency in the expression of MYB and flavonoid synthesis genes [23]. Our findings indicate that genes responsive to JA and OPDA are upregulated when CO_2_ is removed from the environment (Figure 7C,D). The upregulation of JA-responsive genes could be triggered by an increase in the production of ROS due to the over-reduction of the PETC, which leads to the synthesis of JA [81,82]. JA then induces the expression of genes by activating the R2R3-MYB/bHLH/WD40 complex, which induces the expression of flavonoid synthesis genes. Flavonoids, being effective scavengers of ROS, can then protect the plant from oxidative stress.

### 4.3. PSI Photoinhibition Changes the Expression of Nuclear Genes Involved in Iron Homeostasis, Light Signaling, and PSI-Acceptor-Side Metabolism

PSI-PI treatment induced the differential expression of 59 genes independently of the CO_2_ concentration (Table S1) and 200 genes in three out of the four differential CO_2_ treatments applied in this study (Figure 5). Interestingly, the leaves exposed to the PSI-PI treatment at atmospheric CO_2_ revealed the highest number of DEGs (810 genes in total) (Figure 5), indicating that, in total, over 1000 genes responded specifically to the PSI-PI treatment. 

PSI-PI treatment induced 24 genes involved in plant Fe homeostasis (Table 2), which is consistent with damage to FeS clusters during PSI photoinhibition [12,66]. Although the PSI-PI treatment does not lead to the degradation of PSI subunits during illumination [28], the damage to FeS clusters may induce the release of Fe from PSI complexes, leading to excess-Fe stress in the chloroplast. Twelve genes induced by PSI photoinhibition are involved in Fe uptake into the cell and cell organelles (FROs, VTLs), Fe cofactor transport (NEET, cHBPs), and Fe storage (FERs) (recent review by Sági-Kazár et al. [44]). FERRIC REDUCTION OXIDASE (FRO) proteins are membrane proteins that catalyze the reduction of Fe^+3^ to Fe^+2^ and thereby facilitate the Fe uptake across the plasma and organelle membranes [43,83]. FRO7 is a part of an iron translocon that transports Fe to the chloroplast [83], and VACUOLAR IRON TRANSPORTERs (VTR) are involved in Fe transport to the vacuole [44]. The majority of Fe in leaves is incorporated into heme and FeS clusters in organelles. FeS clusters are also required in the cytosol, and both chloroplastic and mitochondrial NEET proteins have been proposed to deliver 2Fe-2S clusters from organelles to cytosolic proteins [84]. The cHBPs carry hemes in the cytoplasm, although their physiological role is still unknown. *FER* genes are induced by Fe excess in plants but also by other stresses, including HL stress (Figure S6) [41,62,85]. Accordingly, the genes involved in the induction and the co-expression network of Fe deficiency were highly repressed in PSI-PI-treated leaves (Table 2) [86,87,88].

**Figure 8:**
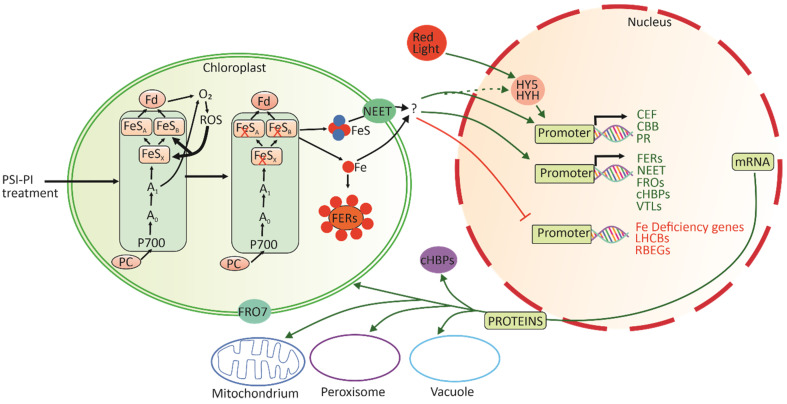
Scheme for the chloroplast retrograde signaling pathway controlling nuclear gene expression in PSI-photoinhibited leaves. PSI-PI treatment induces the accumulation of electrons on the acceptor side of PSI, which increases ROS production, followed by damage to FeS centers in PSI (FeS_A_, FeS_B_, FeS_X_). Damage to FeS centers may lead to the release of iron. Iron is scavenged by ferritins (FERs), but when the iron content exceeds the binding capacity of FERs, the rise in iron concentration initiates a retrograde signaling cascade, activating iron homeostasis genes responding to excess iron and repressing iron-deficiency genes. The signal also activates the genes encoding PSI-acceptor-side pathways, cyclic electron flow (CEF), the Calvin–Benson–Bassham (CBB) cycle, and photorespiration (PR). Alternatively, FeS centers may be released from the damaged PSI and transported to the cytosol via NEET protein, transferring the signal from the chloroplast to the nucleus. The mediators of the signaling cascade are unknown. Furthermore, the expression of CEF, CBB cycle, and PR genes may also be regulated by a combination of red-light-induced transcription factors like HY5 and HYH and Fe signaling.

FERs bind iron and sequester it in an inert form, thereby protecting cells from oxidative damage by the Fenton reaction [44]. Indeed, the degradation of PSI complexes in *Chlamydomonas* has been shown to induce the accumulation of FER proteins [89], suggesting that an increased risk of Fe release from PSI proteins activates *FER* gene expression. Consistently, in the *pgr5* mutant, which suffers from severe PSI photoinhibition under HL [90], *FER*s and iron transporter genes are induced by HL illumination [91]. We postulate that the damage to PSI induces the release of Fe from PSI FeS clusters in the chloroplast and that such an excess of Fe in turn initiates a signal from the chloroplast to the nucleus (Figure 8). This signal upregulates the expression of genes involved in Fe homeostasis, whereas the genes known to be induced by Fe deficiency in plants, are simultaneously repressed by PSI photoinhibition (Table 2; Figure 8).

PSI-PI treatment also targeted a group of light-signaling regulators by inducing or repressing the expression of the respective genes (Table 3). The *HY5*, *HYH*, *COL2,* and *ARF* genes encode nuclear-located transcription factors that activate light-responsive genes. The influence of the master transcription factor HY5 on the activation of light-regulated genes during photomorphogenesis is well established (for reviews, see, e.g., [48,49]), while much less is known about the function of HY5 and HYH in later stages of leaf development and in mature leaves. HY5 has been reported to be expressed in various organs of adult tomato plants, including roots [92], and its expression has been shown to increase in response to high [93] and UV-B light [94]. HY5 is also linked to several hormone- and stress-signaling cascades [16,48,49,95], and it has been suggested to act downstream of chloroplast retrograde signaling to the nucleus [16,96,97]. Our transcriptomic data indicate that HY5 and HYH expression can also be induced by exposing mature leaves to fluctuating light, which causes PSI photoinhibition (Table 3). Chloroplast-localized SIG5 is a member of the plant sigma factor family, and this gene is induced by several stresses [98]. It controls the activity of plastid-encoded RNA polymerase (PEP), which is responsible for the transcription of plastid photosynthetic genes [99]. The upregulation of *SIG5* in PSI-photoinhibited leaves suggests that stress not only affects nuclear gene expression but also affects plastid gene expression via the regulation of PEP polymerase. 

In addition to the induction of light-signaling transcription factors, PSI-PI treatment significantly reduced the expression of genes encoding suppressors of photomorphogenesis (PIF2/PIL1, PIF6/PIL2) and photosynthetic gene expression (RPGEs) (Table 3). GOLDEN2-LIKE transcription factors (GLKs) are potential targets of RPGE regulation. GLKs function as dimers, and they primarily activate nuclear genes encoding photosynthesis proteins by binding to the CCAATC sequence in the promoter region [100]. RPGEs inhibit the binding of GLKs to DNA by specifically interacting with GLK proteins and disrupting GLK dimerization [101]. In our experiment, GLK genes were not differentially expressed, but the significant repression of RPGE genes in PSI-PI-treated leaves compared to GL-treated leaves may release GLK proteins from the inhibitory RPGE complex and allow the post-translational activation of GLKs by dimerization. 

PSI-PI treatment modifies photosynthetic metabolism in chloroplasts, and, accordingly, genes encoding chloroplast proteins were highly represented among the DEGs in PSI-PI-treated leaves (Table S1). A special focus on the differential expression of photosynthetic genes revealed that PSI photoinhibition increased the expression of genes encoding electron acceptors of PSI: CEF, CBB cycle, and photorespiration components (Figure 6). In the future, this finding needs to be confirmed by proteomic analyses. Of the enzymes comprising both photosynthetic and non-photosynthetic isoforms, only the photosynthetic isoforms showed differential expression in PSI-PI-treated leaves compared to GL leaves, emphasizing the specificity of the signal. A photosynthetic DEG profile similar to that induced by the PSI-PI treatment was found only in the leaves exposed to HL at 100 times the intensity of growth light (Figure S7), the light intensity most likely to induce strong PSI photoinhibition in leaves. 

### 4.4. Origin of the Signal(s) Initiating PSI Photoinhibition-Responsive Expression of Nuclear Genes

Three potential sources of the signal(s) initiating the differential expression of nuclear genes during the PSI-PI treatment of leaves can be envisaged. Such predictions, based on the light quality of the PSI-PI treatment, changes in chloroplast redox states during the treatment, and the DEG profiles obtained from treated leaves, include the following options as sources and putative signaling pathways: (i) low-intensity red-light pulses of fluctuating light inducing Phy signaling, (ii) redox imbalance in PETC components and the consequent generation of ROS signaling, and (iii) excess Fe stress in chloroplasts inducing putative retrograde signaling. 

Option (i) Theoretically, the short low-intensity red-light pulses in the fluctuating-light regime could directly promote Phy-mediated regulation of nuclear gene expression, which could explain the higher expression of HY5/HYH transcription factors and the other positive effectors and the concomitant downregulation of specific light signaling repressors in PSI-PI-treated leaves (Table 3). Nevertheless, Phy generally activates the expression of photosynthetic genes under light (Figure S7, dark to light) [17], whereas we observed the selective induction and repression of photosynthetic genes by the PSI-PI treatment (Figure 6). Accordingly, the red-light pulse of the PSI-PI treatment may be responsible for the differential expression of Phy-related transcription factors and repressors but cannot fully explain the expression profiles of photosynthetic genes in PSI-PI-treated leaves. Alternatively, PSI photoinhibition may initiate chloroplast retrograde signal(s) that alter the expression/function of nuclear light-responsive transcription factors and repressors (Figure 8), as recently demonstrated under stresses caused by the dysfunction of chloroplast biogenesis (see recent reviews [15,16,17]). 

Option (ii) PSI-PI treatment over-reduces the redox components between PSI and PSII [28], leading to a redox imbalance between the light and carbon fixation reactions in chloroplasts, which in turn can increase ROS production in thylakoid complexes [90]. Both the redox imbalance and ROS generation have been shown to initiate redox signals from the chloroplast to control the nuclear gene expression. Since the redox imbalance induced by PSI-PI treatment mimics that induced by the HL treatment, we compared the DEGs induced in PSI-PI-treated leaves with recently published transcriptomic data on the induction of photosynthetic genes upon the HL treatment of plants (Figure S6) [62,102]. This analysis revealed that the strongest DEGs present in PSI-PI-treated leaves (Table 2 and Table 3) were expressed in leaves exposed to HL for 3.5 h (Figure S6) [62]. Approximately 70% of the genes upregulated by our PSI-PI treatment were also upregulated by HL, whereas most of the genes downregulated by PSI-PI did not respond to the HL treatment (Figure S6). However, most of the genes encoding CEF, CBB cycle, and photorespiration components were not differentially expressed in response to HL [62,102], contrary to their DEG behavior in the PSI-PI treatment. This suggests that the stress induced by PSI-PI treatment is, at least in part, different from that induced by HL and likely relays additional signals from chloroplasts to the nucleus.

Singlet oxygen and H_2_O_2_ are the major stress-induced ROS produced by photosynthetic light reactions. In mature leaves, photodamaged PSII is a major source of ^1^O_2_ in the chloroplast, while PSI donates electrons to molecular oxygen, leading to the generation of superoxide and H_2_O_2_ [103]. This prompted us to test whether the genes known to be induced by ^1^O_2_ and H_2_O_2_ were upregulated in our experimental setup (Figure 7). Very few of the genes known to be induced by ^1^O_2_ were upregulated upon PSI-PI treatment (Figure 7), consistent with a very weak inhibition of PSII in treated leaves (Figure 2). H_2_O_2_ does not seem to act as a major signal modifying nuclear gene expression, as only two genes (*CAT2* and *FER1*) are strongly upregulated in PSI-PI-treated leaves (Figure 7). It is likely that, despite the imbalanced redox state in the chloroplast, there is no increased production of ROS in leaves with prolonged PSI photoinhibition [90], probably due to the enhanced non-photochemical quenching of energy in damaged PSI [12,28]. Furthermore, the known JA/OPDA-induced genes, whose products have been suggested to mediate thylakoid-initiated retrograde signaling [22,23], were mostly downregulated or did not respond at all to the PSI-PI treatment (Figure 7).

Option (iii) Only one PSI-PI-treatment-specific trigger for retrograde signaling from chloroplasts to the nucleus was possible to be traced based on our experiments and data analyses. PSI-PI treatment induced severe PSI inhibition, which is initiated by damage to the FeS clusters [12,66]. We propose that the release of Fe from the photodamaged PSI complex causes excess-Fe stress in the chloroplast, which initiates a retrograde signal to the nucleus (Figure 8). This chloroplast signal targets the genes that alleviate Fe stress, facilitate Fe transport, and improve the acceptor-side sink capacity of PSI. The production of hydroxyl radicals via the Fenton reaction between H_2_O_2_ and Fe^+2^ [10] may originally initiate the cascade, although OPDA signaling, generally associated with lipid peroxidation by hydroxyl radicals [104], was not particularly activated by the PSI-PI treatment (Figure 7C). Alternatively, FeS centers may be released from the damaged PSI and transported to the cytosol via the NEET protein (Figure 8). Such an effect on nuclear gene expression partially mimics the HL stress, while the effect is stronger and more selective than that observed when only increasing the light intensity (Figure S6). It has been reported that HL alone, without any fluctuating-light treatment, damages the PSI FeS_A_ and FeS_B_ clusters, while under strong PSI photoinhibition, the FeS_X_ cluster is also damaged [12], suggesting that strong PSI photoinhibition induces more severe excess-Fe stress than a moderate increase in light intensity. PSI-PI-treatment- and HL-induced stress distinctively differ from each other by the fact that HL stress also induces the strong photoinhibition of PSII, together with ROS production [105,106], which is not observed in the PSI-PI treatment [90]. A literature search of DEG profiles shows that both PSI-PI and HL treatments induce genes generally associated with excess-Fe stress (Table 2, Figure S7), whereas the well-defined ROS signaling resulting from HL is absent in PSI-PI-treated leaves (Figure 7) [91]. Furthermore, PSI-PI treatment represses Fe-deficiency genes, whereas HL has no effect on the expression of these genes (Figure S6) [62]. Importantly, the PSI-PI treatment also clearly induces the genes encoding sinks for electrons from PSI, whereas the expression of these genes was not specifically altered under HL [62,102]. 

## 5. Conclusions

Taken together (Figure 8), we conclude that PSI photoinhibition results in damage to FeS clusters and the subsequent release of Fe from PSI. Excessive-Fe stress in the chloroplast initiates a novel retrograde signal to modify nuclear gene expression. Such a signaling cascade controls the expression of iron homeostasis genes and probably also affects the expression of CEF components, the CBB cycle, and photorespiration genes. These changes in nuclear gene expression help plants to survive both the actual stress and subsequent recovery periods. 

It was also shown that CO_2_ deprivation activates genes involved in the biosynthesis of flavonoids via the JA/OPDA signaling cascade. Flavonoids are known to alleviate oxidative stress that is induced in the absence of CO_2_ in plant leaves.

## Figures and Tables

**Table 1:** Differential expression of genes involved in flavonoid synthesis in response to CO_2_ deprivation. The log2 fold changes are presented in the columns (left to right): columns 1–3, changes induced by CO_2_ concentrations under constant growth light (GL); columns 4–6, changes induced by CO_2_ concentrations in PSI-PI-treated plants. Fold-change value is bolded when a *p* < 0.05.

				Log2 Fold Change					
Gene ID	Gene Name	Description		0 ppm CO_2_ GL vs. 400 ppm CO_2_ GL	100 ppm CO_2_ GL vs. 400 ppm CO_2_ GL	1000 ppm CO_2_ GL vs. 400 ppm CO_2_ GL	0 ppm CO_2_ PSI-PI vs. 400 ppm CO_2_ PSI-PI	100 ppm CO_2_ PSI-PI vs. 400 ppm CO_2_ PSI-PI	1000 ppm CO_2_ PSI-PI vs. 400 ppm CO_2_ PSI-PI
AT5G42800	*DFR*	Dihydroflavonol 4-reductase	Endoplasmic reticulum	**10.52**	−0.72	5.72	**8.55**	0.06	−0.22
AT5G17220	*GSTF12*	Glutathione S-transferase phi 12	Cytosol	**8.08**	−0.38	4.49	**6.8**	−0.18	0.6
AT5G54060	*UF3GT*	UDP-glucose:flavonoid 3-o-glucosyltransferase	Chloroplast	**7.97**	−2.74	4.28	**6.9**	0.25	1.51
AT3G29590	*AT5MAT*	HXXXD-type acyl-transferase family protein	Chloroplast	**7.71**	−0.7	3.33	**5.83**	−0.93	1.32
AT4G22880	*LDOX*	Leucoanthocyanidin dioxygenase	Nucleus	**7.03**	−0.43	2.2	**6.82**	−0.2	0.64
AT5G13930	*CHS*	Chalcone and stilbene synthase family protein	cytosol	**5.99**	−0.33	1.89	**4.54**	0.58	1.16
AT5G05270	*CHIL*	Chalcone-flavanone isomerase family protein	Chloroplast	**2.9**	0.89	0.59	**1.76**	1.37	0.63
AT1G66390	*MYB90*	Myb domain protein 90	Nucleus	**8.19**	0.3	6.25	**5.94**	−3.14	−1.52
AT4G09820	*TT8*	Basic helix-loop-helix (bHLH) DNA-binding superfamily protein	Cytosol	**6.11**	−0.42	3.89	**5.24**	−0.39	1.47
AT5G41315	*GL3*	Basic helix-loop-helix (bHLH) DNA-binding superfamily protein	Nucleus	**4.31**	1.92	2.88	**3.17**	−0.13	1.67
AT1G66370	*MYB113*	Myb domain protein 113	Nucleus	**4.23**	−2.88	0.73	**5.21**	−1.68	−1.4
AT2G37260	*TTG2*	WRKY family transcription factor family protein	Nucleus	**3.14**	1.66	1.3	**1.9**	0.75	0
AT1G56650	*MYB75*	Production of anthocyanin pigment 1	Nucleus	**3.09**	−1.04	1.34	**2.99**	−1.21	0.75
AT1G66380	*MYB114*	Myb domain protein 114	Nucleus	**3.01**	−3.1	0.87	**4.97**	0.85	2.17
AT2G47190	*MYB2*	Myb domain protein 2	Nucleus	**2.66**	0.31	0.4	**1.09**	−0.38	0.48
AT5G56840	*AT5G56840*	Myb-like transcription factor family protein	Nucleus	**2.2**	0.18	1.1	**1.1**	0.41	0.55
AT3G06490	*MYB108*	Myb domain protein 108	Nucleus	**1.94**	−0.32	0.73	**2.25**	−0.09	0.42
AT1G52880	*NAM*	NAC (No Apical Meristem) domain transcriptional regulator superfamily protein	Nucleus	**1.69**	0.56	0.59	**1.35**	0.11	−0.1
AT1G77450	*NAC032*	NAC domain-containing protein 32	Nucleus	**1.53**	0.6	−0.03	**1.89**	0.12	0.22
AT2G16720	*MYB7*	Myb domain protein 7	Nucleus	**1.34**	−0.23	0.38	**2.03**	0.08	0.78

**Table 2:** Differential expression of iron metabolism genes in leaves exposed to PSI photoinhibition (PSI-PI) treatment. The fold-change columns show log2 fold changes induced by PSI-PI treatment versus growth light (GL) treatment at different CO_2_ concentrations indicated in the table. Fold-change value is bolded when *p* < 0.05.

				Log2 Fold Change			
Gene ID	Gene Name	Description	Localization	0 ppm CO_2_ PSI-PI vs. 0 ppm CO_2_ GL	100 ppm CO_2_ PSI-PI vs. 100 ppm CO_2_ GL	400 ppm CO_2_ PSI-PI vs. 400 ppm CO_2_ GL	1000 ppm CO_2_ PSI-PI vs. 1000 ppm CO_2_ GL
AT3G56090	*FER3*	Ferritin 3	Chloroplast	**2.69**	**1.93**	**2.34**	**3.05**
AT5G01600	*FER1*	Ferretin 1	Chloroplast	**2.21**	**1.67**	**2.51**	**4.69**
AT5G51720	*NEET*	2Fe−2S cluster binding protein	Chloroplast	**1.91**	**1.14**	**1.38**	**1.97**
AT2G40300	*FER4*	Ferritin 4	Chloroplast	**1.37**	**0.86**	**1.15**	**2.15**
AT3G49160	*AT3G49160*	Expression of the gene is downregulated in the presence of paraquat, an inducer of photooxidative stress. Downregulated by Fe deficiency.	Chloroplast?	**1.32**	**1.67**	**1.64**	**3.52**
AT5G17170	*ENH1*	Enhancer of SOS3-1/rubredoxin family protein	Chloroplast?	**1.27**	**1.02**	**1.36**	**1.55**
AT1G17100	*cHBP1*	Cytosolic heme-binding protein 1	Cytoplasm?	**1.04**	**1.39**	**1.81**	**1.76**
AT1G01590	*FRO1*	Ferric reduction oxidase 1	Plasma membrane	1.02	0.83	**2.31**	**1.1**
AT1G76800	*VTL2*	Vacuolar iron transporter (VIT) family protein	Vacuolar membrane	**0.76**	**1.09**	**0.87**	**1.61**
AT5G49740	*FRO7*	Ferric reduction oxidase 7	Chloroplast envelope	**0.66**	**1.13**	**1.83**	**1.97**
AT5G49730	*FRO6*	Ferric reduction oxidase 6	Plasma membrane	0.64	0.66	**1.88**	**1.46**
AT1G78450	*cHBP3*	Cytosolic heme-binding protein 3	Cytoplasm?	**0.63**	**0.77**	**1.56**	**0.89**
AT1G21140	*VTL1*	Vacuolar iron transporter (VIT) family protein	Vacuolar membrane	0.59	**1.07**	**0.57**	**1.49**
AT2G37970	*cHBP2*	Cytosolic heme-binding protein 2	Cytoplasm?	0.25	**0.78**	**1.07**	**1.35**
AT2G28160	*bHLH029*	basic helix-loop-helix, FER-like regulator of iron uptake	Nucleus	−0.6	**−0.86**	**−1.05**	**−0.96**
AT5G04150	*BHLH101*	Basic helix-loop-helix (bHLH) DNA-binding superfamily protein, response to Fe deficiency	Nucleus	**−2.01**	−1.82	−0.67	**−3.51**
AT3G56360	*AT3G56360*	Hypothetical protein, response to Fe deficiency	Plastid?	**−2.44**	**−1.99**	**−2.43**	**−3.03**
AT2G30766	*FEP1*	FE-uptake-inducing peptide 1, response to Fe deficiency	Cytoplasm or nucleus	**−3.28**	**−3.66**	**−4.49**	**−4.92**
AT1G47395	*FEP2*	Fe-uptake-inducing peptide 2, response to Fe deficiency	Cytoplasm or nucleus?	−3.7	−1.57	−4.33	**−4.82**
AT1G13609	*DEFL*	Defensin-like (DEFL) family protein, response to Fe deficiency	?	−4.04	−3.1	−4.54	**−8.96**
AT2G14247	*IRP3*	Iron-responsive protein 3	Chloroplast	−4.45	−4.05	**−6.09**	**−8.79**
AT5G05250	*AT5G05250*	Hypothetical protein, response to Fe deficiency	?	**−5.1**	**−5.9**	**−6.36**	**−6.31**

**Table 3:** Differential expression of light-signaling genes in leaves exposed to PSI photoinhibition (PSI-PI) treatment. The fold-change columns show log2 fold changes induced by PSI-PI treatment versus growth light (GL) treatment at different CO_2_ concentrations indicated in the table. Fold-change value is bolded when *p* < 0.05.

				Log2 Fold Change			
Gene ID	Gene Name	Description	Localization	0 ppm CO_2_ PSI-PI vs. 0 ppm CO_2_ GL	100 ppm CO_2_ PSI-PI vs. 100 ppm CO_2_ GL	400 ppm CO_2_ PSI-PI vs. 400 ppm CO_2_ GL	1000 ppm CO_2_ PSI-PI vs. 1000 ppm CO_2_ GL
AT5G11260	*HY5*	Long hypocotyle 5, bZIP transcription factor	Nucleus	**1.96**	**2.03**	**2.53**	**2.07**
AT3G17609	*HYH*	HY5-homolog	Nucleus	**1.69**	**2.06**	**2.27**	**2.55**
AT3G02380	*COL2*	CONSTANS-like 2	Nucleus	**1.49**	**1.65**	**2.23**	**1.71**
AT2G24540	*AFR*	Attenuated far-red response	Cytoplasm?	**1.36**	**1.9**	**2.29**	**2.09**
AT5G18404	*AT5G18404*	Small protein, response to red or far-red light	Nucleus	**1.36**	**1.06**	**1.66**	**1.14**
AT5G24120	*SIG5*	Sigma factor 5, regulation of plastid genes	Chloroplast	**1.02**	**2.04**	**2.46**	**1.89**
AT3G62090	*PIF6/PIL2*	Phytochrome interacting factor 3-like 2	Nucleus	0.04	−1.39	−0.57	**−1.54**
AT1G10657	*RPGE4*	Repressor of photosynthetic genes 4	Nucleus	−0.06	−0.65	**−1.29**	−0.25
AT3G56710	*SIB1*	Sigma factor binding protein 1	Chloroplast	−0.16	−0.74	**−1.47**	**−0.83**
AT3G55240	*RPEG3*	Repressor of photosynthetic genes 3	Nucleus	**−1.1**	**−2.28**	**−2.38**	**−1.94**
AT5G02580	*RPGE1*	Repressor of photosynthetic genes 1	Nucleus	**−2**	**−2.69**	**−2.39**	**−2.68**
AT2G46970	*PIF2/PIL1*	Phytochrome-interacting factor 3-like 1	Nucleus	**−2.28**	**−2.66**	**−2.5**	**−2.56**

## Data Availability

Raw data files and count datasets generated for this study are stored in NCBI’s Gene Expression Omnibus (GEO) with the accession code GSE242125.

## Supplementary Materials

The following supporting information can be downloaded at https://www.mdpi.com/article/10.3390/antiox12111902/s1: Figure S1. Stomatal aperture in control plant leaves and in leaves treated with PSI photoinhibition light regime for three hours. Figure S2. The number of differentially expressed genes (DEGs) in both control (GL) and PSI-PI-treated plants at indicated CO_2_ concentrations. Figure S3. Localization of the enzymes in Calvin–Benson–Bassham (CBB) cycle and photorespiration pathway in leaves exposed to PSI photoinhibition. Figure S4. Differential expression of genes involved in starch and sucrose metabolism in *Arabidopsis*. Figure S5. Differential expression of NO-responsive genes in leaves exposed to PSI photoinhibition at various CO_2_ concentrations. Figure S6. Comparison of the gene expression in plants exposed to PSI photoinhibition treatment (PSI-PI) with that in plants exposed to high-light (HL) treatment. Figure S7. Differential expression (log2 values) of *Arabidopsis* nuclear genes encoding (A) photosynthetic electron transport chain (PETC) components, including cyclic electron flow (CEF) components, and (B) Calvin–Benson–Bassham (CBB) cycle and photorespiratory components in leaves exposed to chemical or environmental treatments indicated above the columns. Table S1. Differentially expressed genes in *Arabidopsis* thaliana leaves treated with either growth light (GL) or with PSI photoinhibition light regime (PSI-PI) at various CO_2_ concentrations. Table S2. Significantly enriched Gene Ontology (GO) terms among the differentially expressed genes (DEGs) in leaves illuminated under growth light (GL) or with PSI photoinhibition light regime (PSI-PI) at various CO_2_ concentrations. Table S3. The lists of nuclear genes used to construct the heatmaps presented in the article. References [33,60,61,62,107] are cited in the supplementary materials.
