# Supplementary material for: PSI Photoinhibition and Changing CO2 Levels Initiate Retrograde Signals to Modify Nuclear Gene Expression
Source: Antioxidants (Basel). 2023 Oct 24;12(11):1902. doi: 10.3390/antiox12111902 (PMC10669900; doi:10.3390/antiox12111902)
Supplement: Supplementary file 1 [file antioxidants-12-01902-s001.zip › Kilic et al_Supplementary materials_Figures.docx]

**Supplementary Materials**

Mehmet Kılıç, Ville Käpylä, Peter J. Gollan, Eva-Mari Aro, and Eevi Rintamäki (2023). PSI photoinhibition and changing CO_2_ levels initiate retrograde signals to modify nuclear gene expression.

**Supplementary Figures**


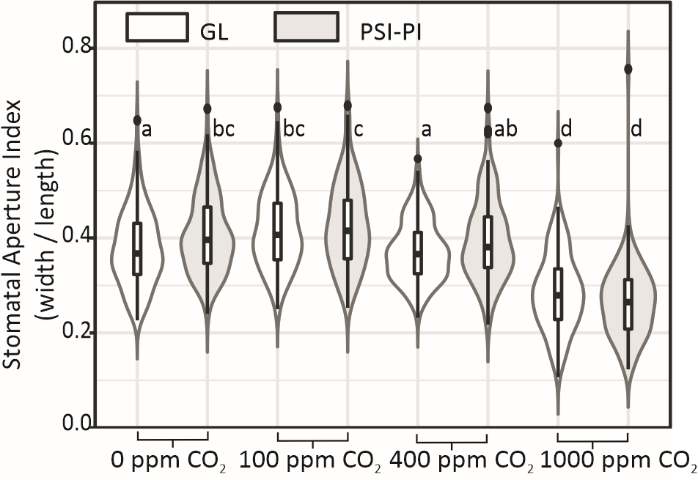


**Figure S1.** Stomatal aperture in control plant leaves (GL; white) and in leaves treated with PSI photoinhibition light regime for three hours (PSI-PI; light grey). After light treatment the abaxial side of leaves was immediately imprinted, and the imprints subsequently examined and imaged under microscope with 40X magnification. Leaf stomatal apertures (n= 25-40 per leaf) were measured using the Fiji image analysis software. Stomatal aperture index (SAI, stomatal width / length) is presented as violin plots with boxplots included inside the data. Bars with different letters indicate significant differences with a p-value < 0.05 (ANOVA, Tukey-HSD).


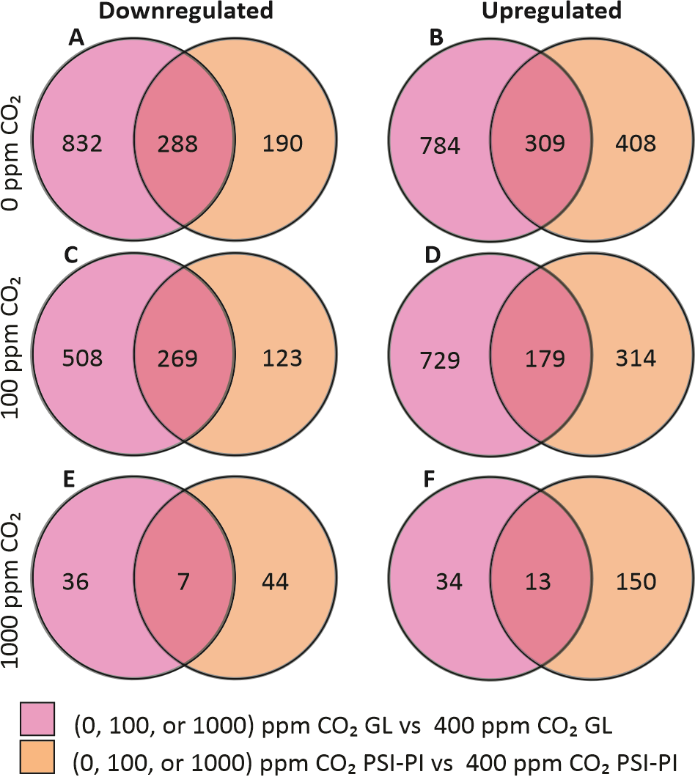


**Figure S2.** The number of the differentially expressed genes (DEGs) both in control (GL) and PSI-PI-treated plants under indicated CO_2_ concentrations. Plants were treated either with growth light (GL, pink) or with PSI photoinhibition light regime (PSI-PI, orange) under CO_2_ concentrations of 0 ppm, 100 ppm, 400 ppm, and 1000 ppm. Venn diagrams were constructed by comparison of differentially expressed genes; **(A)** downregulated under 0 ppm CO_2_, **(B)** upregulated under 0 ppm CO_2_, **(C)** downregulated under 100 ppm CO_2_, **(D)** upregulated under 100 ppm CO_2_, **(E)** downregulated under 1000 ppm CO_2_, **(F)** upregulated under 1000 ppm CO_2_, at GL without PSI-PI treatment (pink) or in leaves treated with PSI-PI (orange) versus corresponding treatment at atmospheric 400 ppm CO_2_. The genes with -1 ≥ log2 ≥ 1 fold change and a p-value < 0.05 were included in the figure. The list of the genes is presented in Table S1.

**
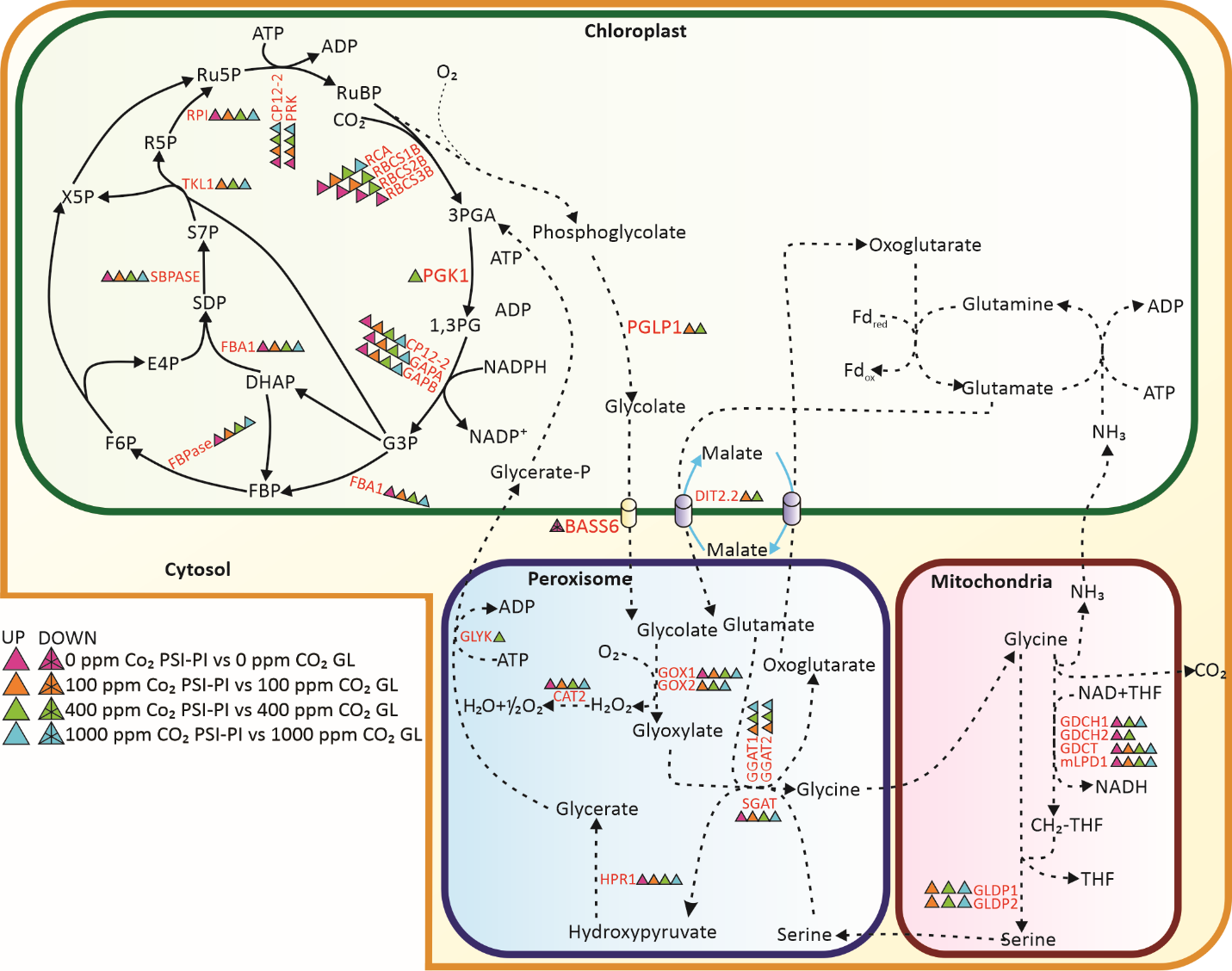
**

**Figure S3.** Localization of the enzymes in Calvin-Benson-Basham (CBB) cycle and photorespiration pathway in leaves exposed to PSI photoinhibition. The leaves were treated in growth light (GL) or exposed to PSI photoinhibition light regime (PSI-PI) treatment under various CO_2_ concentrations. The genes with -0.5 ≥ log2 ≥ 0.5 fold change and a p-value < 0.05 were collected in the figure. CBB cycle reactions are shown with solid black arrows, while photorespiration reactions are shown with black dashed line. Cyan arrows indicate malate valve. Non-crossed shapes indicate up regulation and crossed shapes indicate down regulation of the gene expression. Full name of the genes, see Table S3.

**
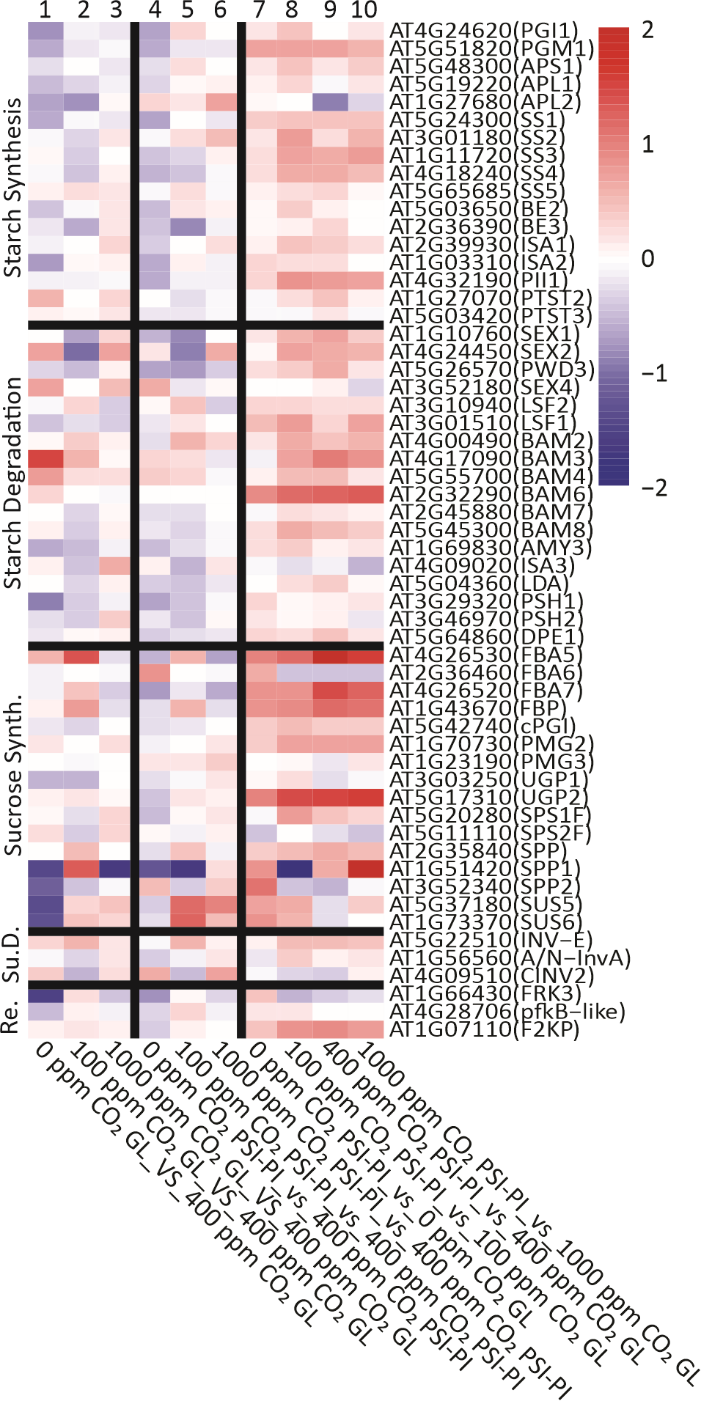
**

**Figure S4.** Differential expression of genes involved in starch and sucrose metabolism of Arabidopsis. The heatmap shows log2 values of differentially expressed genes in plants exposed to growth light (GL) and PSI photoinhibition light regime (PSI-PI) for three hours under various CO_2_ concentrations. The treatments compared in the columns are indicated under the figure. The gene lists for starch synthesis and degradation sucrose synthesis and degradation (Ashburner et al., 2000; Mi et al., 2019) including also regulatory genes, are collected manually and presented in Table S3. The columns: 1-3; changes induced by CO_2_ concentrations at growth light, 4-6; changes induced by CO_2_ concentrations in PSI-PI treated plants, 7-10; changes induced by PSI-PI treatment. Su.D.: Sucrose degradation, Re.: Regulation.


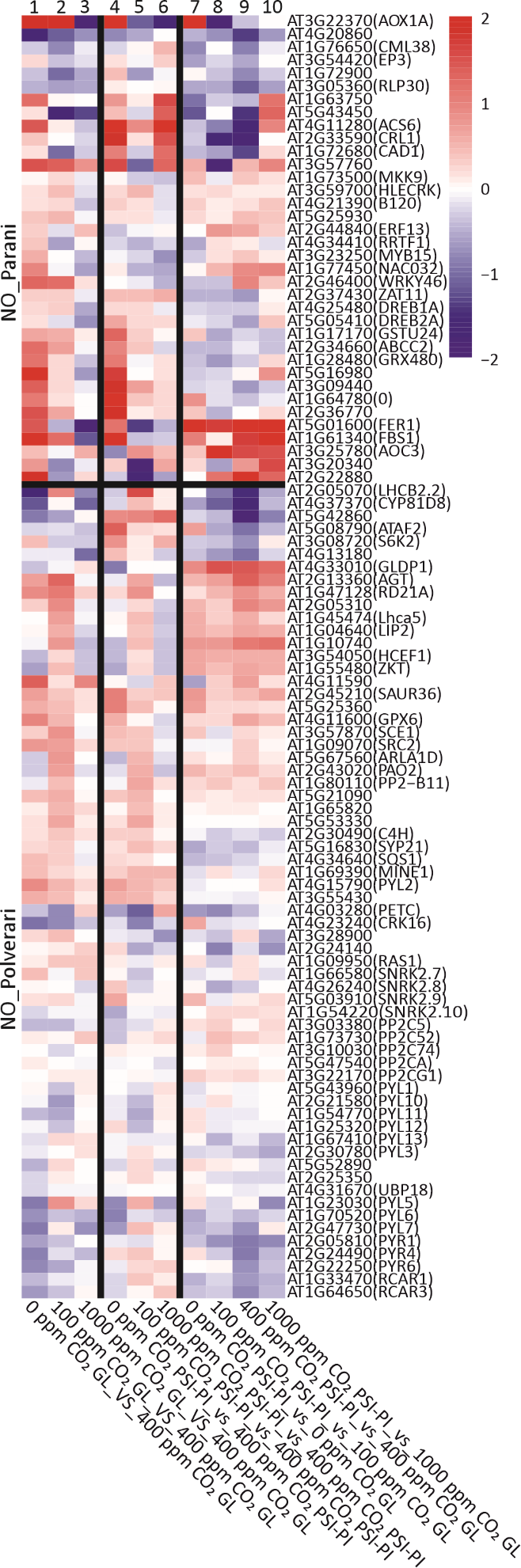


**Figure S5.** Differential expression of NO responsive genes in leaves exposed to PSI photoinhibition at various CO_2_ concentrations. The genes reported to be upregulated in response to nitric oxide (NO) treatment (Parani et al., 2004; Polverari et al., 2003) were included in the analysis (Table 3). The heatmap shows log2 values of differentially expressed genes in plants exposed to PSI photoinhibition for three hours under various CO_2_ concentrations. The treatments compared in the columns are indicated under the figure. The columns: 1-3; changes induced by CO_2_ concentrations at constant growth light, 4-6; changes induced by CO_2_ concentrations in PSI-PI treated plants, 7-10; changes induced by PSI-PI treatment.

**
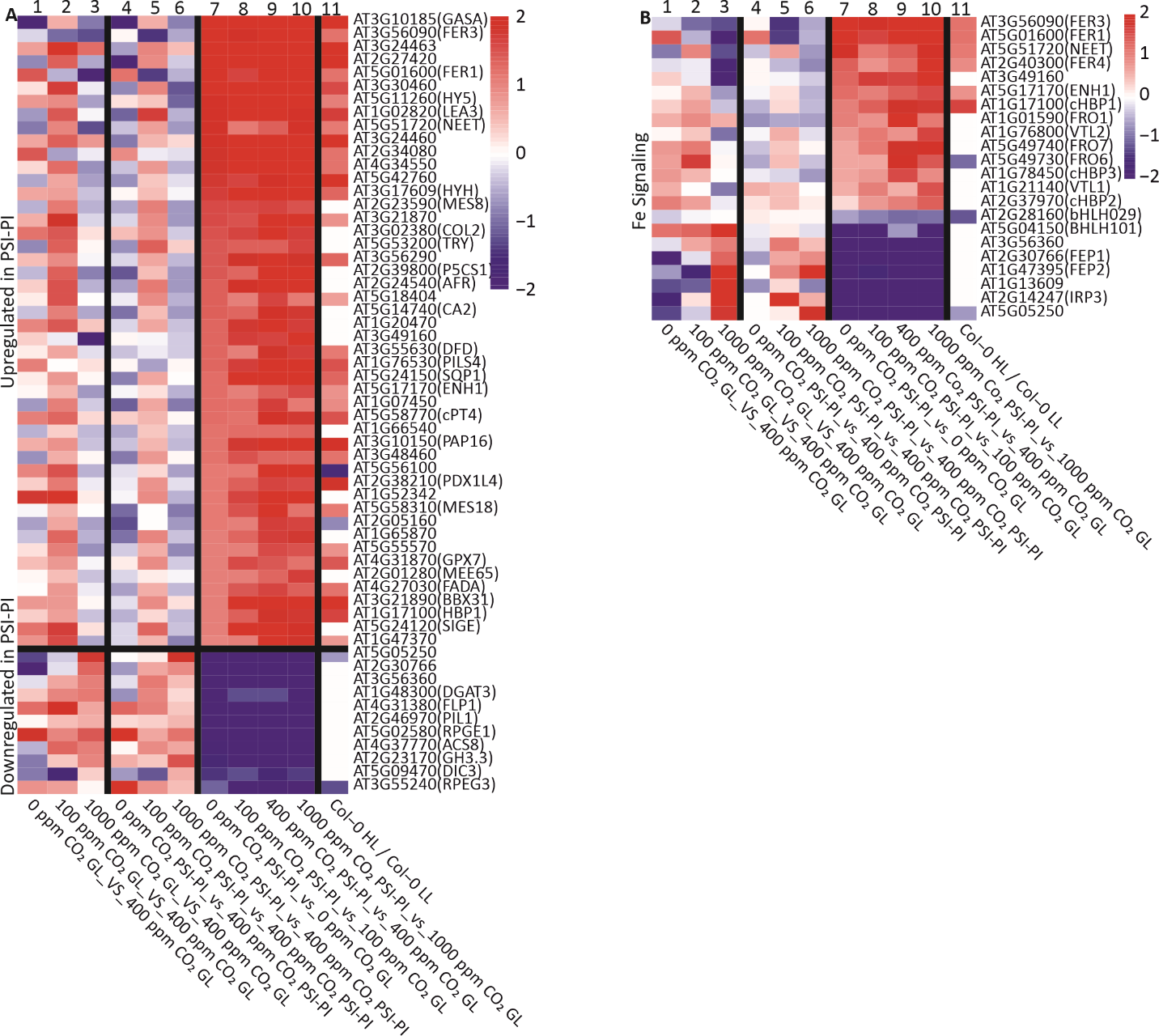
**

**Figure S6.** Comparison of the gene expression in plants exposed to PSI photoinhibition treatment (PSI-PI) with the plants exposed to high light (HL) treatment. **(A)** Expression of the genes that were strongly differentially expressed in leaves after PSI-PI treatment (columns 1 to 10, Table S1) or in leaves exposed to HL for 3.5 hours (column 11). **(B)** Expression of the Fe homeostasis genes that were strongly differentially expressed in leaves after PSI-Pi treatment (Table 2) (columns 1 to 10) or in leaves exposed to HL for 3.5 hours (column 11). The HL gene expression data has been taken from Alvarez-Fernandez et al. (2021), where Arabidopsis plants grown under 150 μmol photons m^-2^ sec^-1^ (LL) were exposed to 1100 μmol photons m^-2^ sec^-1^ (HL) for 3.5 hours. The treatments compared in the columns are indicated under the figure. The columns: 1-3; Log2 fold changes induced by CO_2_ concentrations at constant growth light, 4-6; Log2 fold changes induced by CO_2_ concentrations in PSI-PI treated plants, 7-10; Log2 fold changes induced by PSI-PI treatment, 11; Log2 fold changes induced by HL (Col-0 HL/Col-0 LL).

**
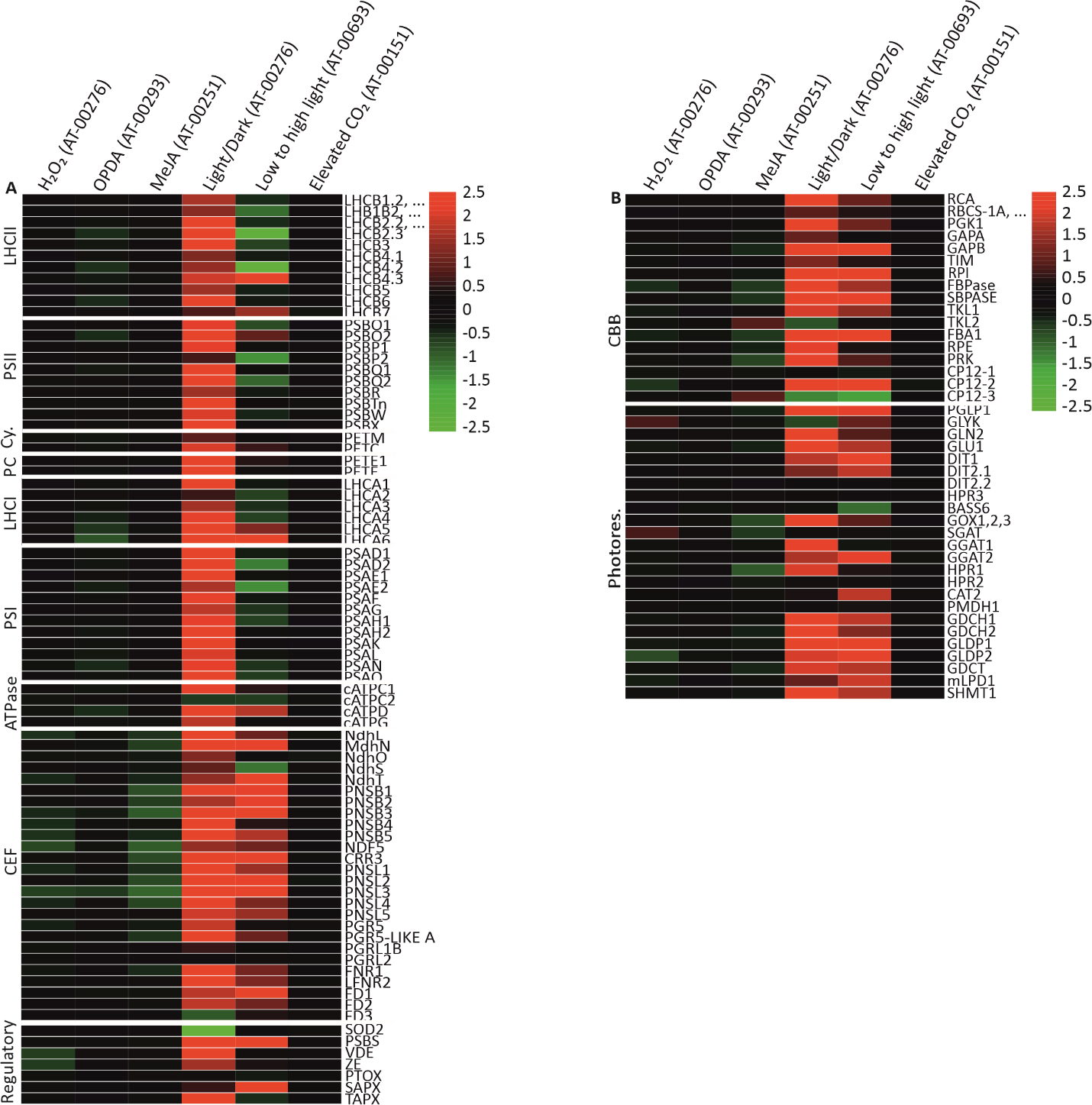
**

**Figure S7.** Differential expression (log2 values) of Arabidopsis nuclear genes encoding **(A)** photosynthetic electron transport chain (PETC) including cyclic electron flow (CEF) components, and **(B)** Calvin-Benson-Basham (CBB) cycle and photorespiratory components in leaves exposed to chemical or environmental treatments indicated above the columns. The experiments tested are indicated by the accession number in Genevestigator database. H_2_O_2_ study **(AT-00185):** Col-0 seedlings, treated with 20 mM hydrogen peroxide for 1 hour versus non treated seedlings. 12-oxo-phytodienoic acid (OPDA) study (**AT-00293):** Col-0 plant samples, treated with OPDA for 4h versus Col-0 plant samples treated with 0.5% methanol in water for 4h. Methyl jasmonate (MeJA) study (**AT-00251):** Col-0 samples treated with 0.5% solution of MeJA (6h) versus mock (6h) treated Col-0 samples. Light/Dark study **(AT-00276):** seven-day-old light grown wild type (Col-0) seedlings versus seven-day-old dark grown wild type (Col-0) seedlings. Low to high light study (**AT-00693):** Rosette samples of Col-0 plants grown on soil for 21 day under short day standard intensity light conditions (100 µmol m-2 s-1) were shifted to low light (8 µmol m-2 s-1) for 10 days, and then shifted to high light (800 µmol m-2 s-1) for 6h versus rosette samples of Col-0 plants grown like described above but kept at low light (8 µmol m-2 s-1) for 6 hours . Elevated CO_2_ study (**AT-00151):** Mature leaf samples from four-week-old Col-0 plants were treated with elevated CO_2_ (750 ppm) versus mature leaf samples from four-week-old Col-0 plants treated with ambient CO_2_ for 24h.

**References**

Alvarez-Fernandez, R., Penfold, C. A., Galvez-Valdivieso, G., Exposito-Rodriguez, M., Stallard, E. J., Bowden, L., Moore, J. D., Mead, A., Davey, P. A., Matthews, J. S. A., Beynon, J., Buchanan-Wollaston, V., Wild, D. L., Lawson, T., Bechtold, U., Denby, K. J., & Mullineaux, P. M. (2021). Time-series transcriptomics reveals a BBX32-directed control of acclimation to high light in mature *Arabidopsis* leaves. *The Plant Journal*, *107*, 1363–1386. https://doi.org/10.1111/TPJ.15384

Ashburner, M., Ball, C. A., Blake, J. A., Botstein, D., Butler, H., Cherry, J. M., Davis, A. P., Dolinski, K., Dwight, S. S., Eppig, J. T., Harris, M. A., Hill, D. P., Issel-Tarver, L., Kasarskis, A., Lewis, S., Matese, J. C., Richardson, J. E., Ringwald, M., Rubin, G. M., & Sherlock, G. (2000). Gene ontology: tool for the unification of biology. *Nature Genetics*, *25*, 25–29. https://doi.org/10.1038/75556

Mi, H., Muruganujan, A., Ebert, D., Huang, X., & Thomas, P. D. (2019). PANTHER version 14: more genomes, a new PANTHER GO-slim and improvements in enrichment analysis tools. *Nucleic Acids Research*, *47*, D419–D426. https://doi.org/10.1093/NAR/GKY1038

Parani, M., Rudrabhatla, S., Myers, R., Weirich, H., Smith, B., Leaman, D. W., & Goldman, S. L. (2004). Microarray analysis of nitric oxide responsive transcripts in *Arabidopsis*. *Plant Biotechnology Journal*, *2*, 359–366. https://doi.org/10.1111/J.1467-7652.2004.00085.X

Polverari, A., Molesini, B., Pezzotti, M., Buonaurio, R., Marte, M., & Delledonne, M. (2003). Nitric oxide-mediated transcriptional changes in *Arabidopsis thaliana*. *Molecular Plant-Microbe Interactions*, *16*, 1094–1105. https://doi.org/10.1094/MPMI.2003.16.12.1094
